# Evaluation of a pediatric post-acute sequelae of SARS-CoV-2 index score

**DOI:** 10.3389/fped.2025.1628826

**Published:** 2025-09-10

**Authors:** Frederick Dun-Dery, Jianling Xie, Kathleen Winston, Brett Burstein, Vikram Sabhaney, Jason Emsley, Jocelyn Gravel, April Kam, Darcy Beer, Roger Zemek, Ahmed Mater, Robert Porter, Gabrielle Freire, Naveen Poonai, Simon Berthelot, Anne Moffatt, Andrew Dixon, Deepti Reddy, Marina Salvadori, Stephen B. Freedman, Pediatric Emergency Research Canada (PERC) COVID Study Group, Samina Ali

**Affiliations:** ^1^ Department of Pediatrics, Cumming School of Medicine, University of Calgary, Calgary, AB, Canada; ^2^ Section of Pediatric Emergency Medicine, Department of Pediatrics, Cumming School of Medicine, University of Calgary, Calgary, AB, Canada; ^3^ Division of Pediatric Emergency Medicine, Department of Pediatrics, Montreal Children’s Hospital, McGill University Health Centre, Montreal, QC, Canada; ^4^ Department of Epidemiology, Biostatistics, and Occupational Health, McGill University, Montreal, QC, Canada; ^5^ Department of Paediatrics, BC Children’s Hospital and BC Children’s Hospital Research Institute, University of British Columbia, Vancouver, BC, Canada; ^6^ Department of Emergency Medicine, IWK Children’s Health Centre and Queen Elizabeth II Health Sciences Centre, Dalhousie University, Halifax, NS, Canada; ^7^ Department of Pediatric Emergency Medicine, Centre Hospitalier Universitaire (CHU) Sainte-Justine, Université de Montréal, Montreal, QC, Canada; ^8^ Division of Emergency Medicine, Department of Pediatrics, McMaster Children’s Hospital, Hamilton, ON, Canada; ^9^ Department of Pediatrics and Child Health, The Children’s Hospital of Winnipeg, Children’s Hospital Research Institute of Manitoba, University of Manitoba, Winnipeg, MB, Canada; ^10^ Department of Pediatrics, University of Ottawa, Children’s Hospital of Eastern Ontario, Ottawa, ON, Canada; ^11^ Department of Emergency Medicine, University of Ottawa, Children’s Hospital of Eastern Ontario, Ottawa, ON, Canada; ^12^ Section of Pediatric Emergency, Department of Pediatrics, Jim Pattison Children’s Hospital, University of Saskatchewan, Saskatoon, SK, Canada; ^13^ Janeway Children’s Health and Rehabilitation Centre, Newfoundland and Labrador Health Services, St John’s, NL, Canada; ^14^ Division of Emergency Medicine, Department of Paediatrics, Hospital for Sick Children, Faculty of Medicine, University of Toronto, Toronto, ON, Canada; ^15^ Department of Paediatrics, Children’s Hospital London Health Sciences Centre, Schulich School of Medicine & Dentistry, London, ON, Canada; ^16^ Children’s Health Research Institute, London, ON, Canada; ^17^ Department of Epidemiology & Biostatistics, Schulich School of Medicine & Dentistry, London, ON, Canada; ^18^ Département de Médecine Familiale et de Médecine d’Urgence, CHU de Québec-Université, Québec, QC, Canada; ^19^ Department of Paediatrics, Kingston Health Sciences Centre, Queen’s University, Kingston, ON, Canada; ^20^ Department of Pediatrics, Stollery Children’s Hospital, University of Alberta, Edmonton, AB, Canada; ^21^ Clinical Research Unit, Children’s Hospital of Eastern Ontario Research Institute, University of Ottawa, Ottawa, ON, Canada; ^22^ Public Health Agency of Canada, Ottawa, ON, Canada; ^23^ Department of Pediatrics, McGill University, Montreal, QC, Canada; ^24^ Sections of Pediatric Emergency Medicine and Gastroenterology, Departments of Pediatrics and Emergency Medicine, Cumming School of Medicine, University of Calgary, Calgary, AB, Canada

**Keywords:** emergency department, index score, post-acute sequelae of SARS-CoV-2, SARS-CoV-2, pediatric

## Abstract

**Objective:**

This study aims to assess the performance of the Researching COVID-19 to Enhance Recovery (RECOVER) initiative's proposed post-acute sequelae of COVID-19 (PASC) index in a cohort of children evaluated for SARS-CoV-2 infection, 6–12 months after exposure.

**Study design:**

We conducted a multicenter, prospective cohort study with 6- and 12-month follow-up in 14 Canadian tertiary-care pediatric emergency departments (EDs) in the Pediatric Emergency Research Canada network. Eligible children were 6 to <18 years of age who were tested for acute SARS-CoV-2 infection. We assessed the score validity and reliability and evaluated the associations between PASC index scores dichotomized using threshold values (≥5.5 for ages 6 to <12 years and ≥5.0 for ages 12 to <18 years) and SARS-CoV-2 infection.

**Results:**

Participants included 785 children, with a median age of 9 years (IQR: 7–13), enrolled between August 2020 and February 2022. Factor analysis identified characteristics that accounted for 32%–40% of variance. Strong correlations were identified between PASC index scores and PedsQL™ and overall health status; Cronbach's *α* ranged from 0.49 to 0.67. Changes in PASC index scores across time points accounted for 71% (6 to <12 years) and 63% (12 to <18 years) of total variance. The proportion of children exceeding PASC index score thresholds did not differ between children positive and negative for SARS-CoV-2 test in the 6 to <12 (25% vs. 22%; aOR: 1.2; 95% CI: 0.6, 2.5) and 12 to <18 (18% vs. 10%; aOR: 2.2; 95% CI: 0.5, 10.4) age groups at 6 months. Similar results were reported at 12 months.

**Conclusions:**

While scores correlated with quality of life and overall health, internal reliability was low to acceptable. The PASC index was not associated with previous SARS-CoV-2 infection.

## Introduction

Post-acute sequelae of SARS-CoV-2 (PASC), or long COVID, remain a critical public health challenge in children and adolescents (1). Multiple definitions exist, most describing symptoms or signs that persist, relapse, or emerge after SARS-CoV-2 infection without alternative explanations (2–4). PASC is particularly challenging to diagnose in children as the presentation differs across age groups and can present with symptoms such as headache (5) and abdominal pain (6), which are commonly seen in otherwise generally healthy children. These challenges have led to highly variable PASC prevalence estimates ranging from 0.5% (7) to 67% (8). Importantly, some studies report limited differences in the prevalence of PASC symptoms between infected and uninfected children (3, 7), questioning the specificity of existing definitions.

A recent study (9) analyzed symptoms reported by children who are infected and uninfected with SARS-CoV-2, identifying those most strongly associated with prior infection, which were included in an index that has been proposed as a research tool to identify children with PASC. Since this PASC index has not been externally evaluated, we assessed the validity and reliability of the index and hypothesized that if the index retained its association with SARS-CoV-2 infection in an external multicenter cohort of children, then it could be considered for clinical use.

## Methods

### Study design and settings

We conducted a secondary analysis of data collected from children enrolled in a multicenter, prospective, longitudinal cohort study conducted between 4 August 2020 and 22 February 2022 (7, 10). Participating institutions (Supplementary Table S1) included 14 Pediatric Emergency Research Canada (PERC) (11) tertiary-care pediatric emergency departments (EDs). Participating sites obtained research ethics board approval, and caregivers provided informed consent; assent was obtained per institutional policy. We followed the Statement for Reporting Studies of Diagnostic Accuracy guidelines (12).

### Participants and recruitment

Children less than 18 years old who underwent testing for SARS-CoV-2 due to symptoms or epidemiologic risk factors for infection (e.g., close contact with positive individuals) were eligible. Specimens were collected at the treating physician's discretion and analyzed per local laboratory standards. To identify potentially eligible children, team members received a list each day of children who had SARS-CoV-2 testing performed. Research assistants attempted to contact by telephone the caregivers of all children who tested positive, followed by contacting those who tested negative. To minimize selection bias, recruitment was standardized across sites by attempting to contact potentially eligible participants consecutively based on the chronological time of specimen collection.

For this sub-study, to align with the age categories included in the PASC derivation study (9), participant eligibility was restricted to those aged 6 to <18 years. Eligible participants completed follow-up surveys, which were added to the protocol on 1 November 2021, at 6 and/or 12 months after their index ED visit; participants enrolled prior to 1 November 2020 were ineligible. These time points were employed to standardize follow-up timing; in the derivation study, follow-up was performed a median of >500 days after infection (9).

### Outcomes and objectives

Research into the pathophysiology of PASC and the conduct of therapeutic trials has been challenging, particularly in children (13). As an extensive evaluation failed to identify any laboratory tests that could serve as useful biomarkers of PASC (14), the Researching COVID-19 to Enhance Recovery (RECOVER) initiative prospectively enrolled and collected symptom data from children infected and uninfected with SARS-CoV-2 to improve our understanding of pediatric PASC (9). Reported symptoms were analyzed, and those most strongly associated with prior infection were included in an index that has been proposed as a research tool to identify children with PASC.

In this study, we sought to assess the psychometric properties of the PASC index through an evaluation of construct validity, which was evaluated via exploratory factor analysis (EFA) and concurrent validity by examining the association between the total PASC index and the PedsQL™-4.0 inventory and caregiver-reported overall health status. Reliability was assessed via Cronbach's *α* and a generalizability (G) study. Finally, to assess the relationship between the PASC score and SARS-CoV-2 infection, we assessed if scores above the RECOVER study's age-specific cut points (6 to <12 years, score ≥5.5; 12 to <18 years, score ≥5) (9) are associated with SARS-CoV-2 test status.

### Data collection

Clinical information from the index ED visit was collected from caregivers and supplemented by medical record review performed 14 days later to identify the index ED SARS-CoV-2 test result and results of additional tests performed. Follow-up data were collected from caregivers 6- and 12-month post-index ED visit using a modified version of the International Severe Acute Respiratory and Emerging Infection Consortium (ISARIC) long-COVID pediatric survey tool (Supplementary Table S2) (15). Self-reported race and ethnicity were collected as disparities exist in PASC prevalence based on these social constructs (16). To standardize data collection, caregivers completed follow-up questionnaires as most surveys were completed during the day when older children and teenagers were unavailable—they were at school or the caregiver was at work.

### Definitions

SARS-CoV-2 status was classified as positive if a nucleic acid test performed on a swab obtained from the nares, nasopharynx, or oral cavity at the index ED visit or during the subsequent 14 days was positive. Non-nucleic acid tests performed at home were infrequently available during the enrollment period, and as such we relied on results of nucleic acid testing. However, all participating sites are in Canada, with provincial health authorities, enabling easy access to results performed in any laboratory or hospital across their province. Participants with negative tests constituted the comparison group. Acute SARS-CoV-2 hospitalization status incorporated events until 14 days after the index ED visit (17). Testing and reporting of variants of concern (VoC) varied by institution and over time. When a VoC or a variant linked to a VoC was identified, that report was used for classification purposes. If VoC testing was not performed or results were inconclusive, the SARS-CoV-2 variant was classified based on the predominant variant circulating at the time of testing (10).

The RECOVER study's PASC index includes 10 symptoms (e.g., trouble with memory or focusing; back, neck, or stomach pain; headache; and fear about specific things) for those 6 to <12 years and 8 symptoms (e.g., change or loss of smell or taste; body, muscle, or joint pain; daytime tiredness; sleepiness or low energy; and tired after walking) for those 12 to <18 years. The maximum achievable scores are 35 and 25 for these age groups, respectively. Follow-up surveys collected data related to all symptoms included in the PASC index; however, the precise language or collection approach varied slightly from the derivation study ( Supplementary Table S3).

Quality of life (QoL) was quantified using age-specific versions of the PedsQL™-4.0 (18), which includes 23 distinct items divided into four subscales that address physical, emotional, social, and academic functioning (19). Caregivers selected survey responses using a five-point Likert scale, ranging from “never” to “almost always” and described the child's well-being in the preceding 7 days. PedsQL™ domain-specific scores were derived by summation of item scores divided by the number of items answered (20). The average of the subdomain scores provided the cumulative PedsQL™ score (19, 20). Participant overall health status was reported by caregivers on a 0- to 100-point visual analog scale.

### Sample size determination

We estimated the sample size in each group (i.e., infected and uninfected) for participants aged 6 to <12 years and 12 to <18 years, to identify a difference between groups in the categorization of participants according to the RECOVER PASC index with 80% power and a two-sided type 1 error of 0.05. Calculations employed age-specific estimates of PASC scores of 20% and 4% for children (6 to <12 years) and 14% and 3% for adolescents (12 to <18 years), among children infected and uninfected with SARS-CoV-2, respectively (9). For the latter group of children, these estimates do not imply they have PASC (as they were uninfected) but rather symptoms yielding scores that meet the threshold proposed for the diagnosis of PASC in SARS-CoV-2 infected individuals. To account for potential site-level clustering effects, as participants were recruited from 14 sites, the sample size was adjusted using a design effect approach. Given that the exact magnitude of the intra-class correlation coefficient (ICC) is unknown, we conducted sample size estimates assuming ICC values of 0.01, 0.05, and 0.10 (21). Using these estimates, the required sample size per group is 67, 76, and 87 participants aged 6 to <12 years, respectively, and 107, 131, and 162 participants aged 12 to <18 years, respectively.

### Statistical analysis

Data were summarized using descriptive statistics, and baseline categorical variables were compared using the *χ*^2^ test, Fisher’s exact test, or Mann–Whitney *U* test, as appropriate. Confidence intervals for the difference between proportions were calculated using the Wald method; the Agresti–Caffo method was used when the event rate was <20% (22). Participants with missing or incomplete outcome data were excluded. All analyses were two-tailed, with significance set at *P* < 0.05. Analyses were performed using SPSS 29.0 (IBM Corp., Armonk, NY, USA) and R version 4.3.3.

Construct validity was assessed using EFA. A two-factor solution was explored through principal axis factor analysis with direct oblimin rotation because we anticipated some correlation between the items included in the index (23). Factor loadings in the resulting pattern matrix reflect the correlation between an item in the index and a specific factor; higher loadings indicate a stronger association with that factor. We used a cutoff of 0.4 to determine whether an item was significantly related to the factor (24). Due to the small sample size (*n* < 50), EFA was not performed for the PASC in the 12- to <18-year-old group at 6 months.

Internal reliability was evaluated using Cronbach's *α* with values ≥0.7 indicative of good internal reliability (25). For generalizability, we conducted a G study on total PASC scores to assess the variance components associated with study sites, assessment time points, and individual participants. This approach provides a framework to evaluate the reliability of a scoring system (26–28), by quantifying the proportion of total variance attributable to different sources. We fitted a random-effects ANOVA model (variance components model) to estimate the variance attributable to study sites, assessment time points, and individual participants. Based on these variance components, we calculated generalizability coefficients to evaluate the relative and absolute reliability of the PASC index. Additionally, we also explored how the generalizability coefficient would improve with additional repeated assessments per participant.

To assess concurrent validity, we calculated Pearson correlation coefficients to examine the relationship between the total PASC index and the PedsQL™-4.0 inventory and caregiver-reported overall health reported on a 0- to 100-point scale. To assess the relationship between exposure to COVID infection, PASC index scores were calculated and classified. To estimate the odds of having a score exceeding the threshold in relation to SARS-CoV-2 test status, we applied logistic regression using generalized estimating equation (GEE) models, adjusting for clustering by site and the presence of chronic conditions at baseline. Separate GEE models were used for each age and survey time point subgroup. As several of the measures included in the PASC index scores include measures from the PedsQL™-4.0 inventory which includes a response option of “sometimes” (Supplementary Table S3), a sensitivity analysis was conducted with this option categorized as the symptom being “absent.”

## Results

### Participant characteristics

**Figure 1:**
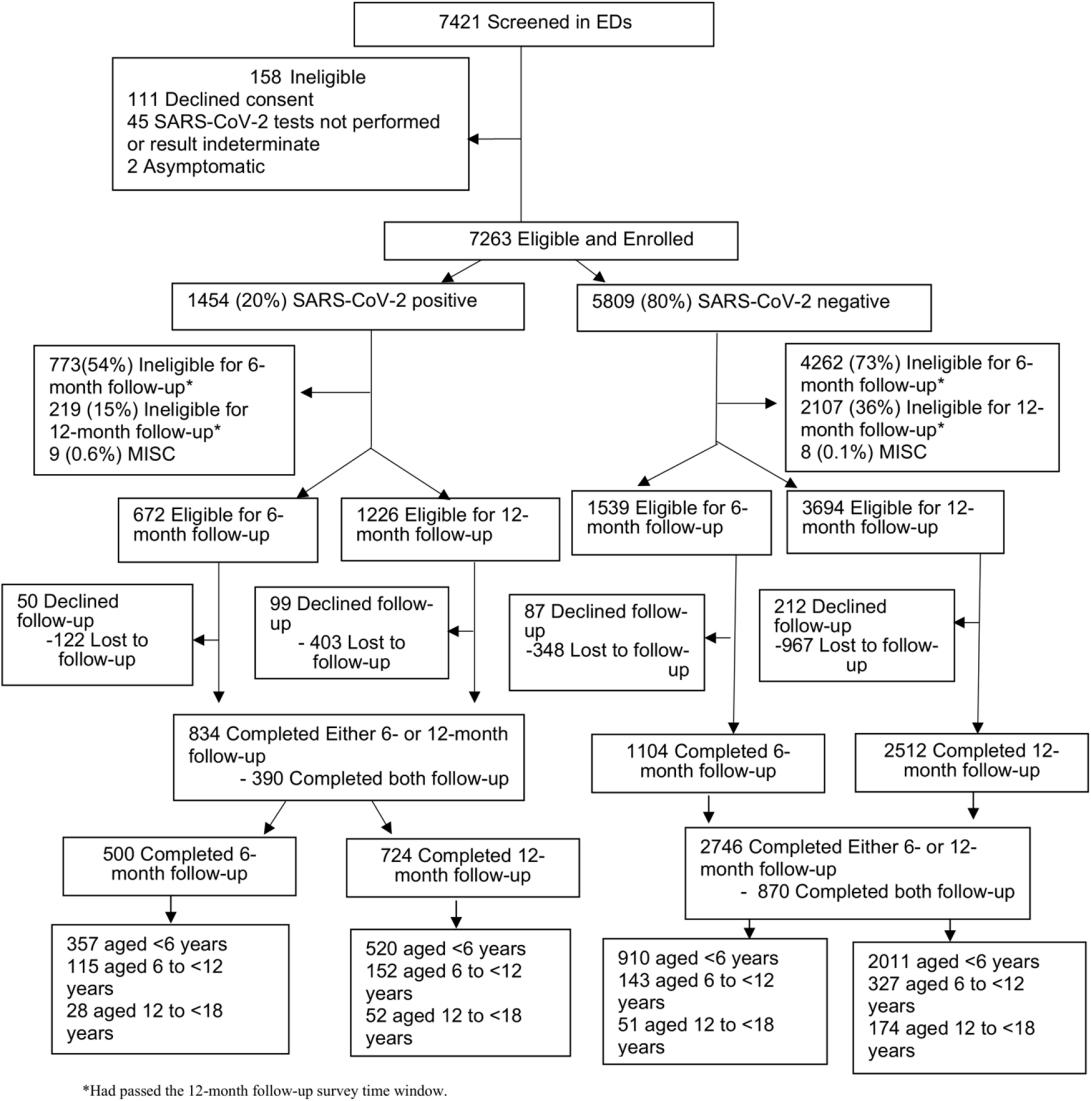
Flow diagram of study participant recruitment, SARS-CoV-2 test status, and completion of 6- and 12-month follow-up surveys.

A total of 785 eligible participants completed either or both follow-up time points (Figure 1). There were no clinically significant differences between those who were and were not lost to follow-up based on SARS-CoV-2 classification (Supplementary Table S4). The median age of the participants was 9 years (IQR: 7, 13), 51.5% (*N* = 401) were male, 46.4% (*N* = 364) self-identified as White, 86.5% (*N* = 679) were discharged from the ED at the index visit, and 1.7% (*N* = 13) were asymptomatic at the time of testing (Table 1).

**Table 1:** Baseline demographics stratified by SARS-CoV-2 test outcome and age groups, for all participants who completed the 6- or 12-month follow-up surveys.

Variable	All	Age 6 to <12 years		Age 12 to <18 years	
		SARS-CoV-2 negative	SARS-CoV-2 positive	SARS-CoV-2 negative	SARS-CoV-2 positive
	*n* = 785	*n* = 360	*n* = 176	*n* = 189	*n* = 60
Age, years, median (IQR)	9.0 (7.0, 13.0)	8.0 (6.0, 9.0)	8.0 (7.0, 10.0)	14.0 (13.0, 16.0)	14.0 (13.0, 16.0)
Sex, male, *n* (%)	401 (51.5)	199 (55.3)	95 (54.0)	75 (39.7)	32 (53.3)
Race, *n* (%)					
Black	51 (6.5)	17 (4.7)	23 (13.1)	5 (2.6)	6 (10.0)
East Asian	31 (3.9)	18 (5.0)	7 (4.0)	6 (3.2)	0 (0)
Indigenous	25 (3.2)	8 (2.2)	3 (1.7)	12 (6.3)	2 (3.3)
Latin American	34 (4.3)	22 (6.1)	7 (4.0)	4 (2.1)	1 (1.7)
Middle Eastern	89 (11.3)	35 (9.7)	26 (14.8)	17 (9.0)	11 (18.3)
Multiracial	103 (13.1)	61 (16.9)	21 (11.9)	16 (8.5)	5 (8.3)
South Asian	48 (6.1)	19 (5.3)	15 (8.5)	7 (3.7)	7 (11.7)
Southeast Asian	26 (3.3)	12 (3.3)	8 (4.5)	5 (2.6)	1 (1.7)
White	364 (46.4)	164 (45.6)	58 (33.0)	116 (61.4)	26 (43.3)
Race missing or unspecified	14 (1.8)	4 (1.2)	8 (4.5)	1 (0.5)	1 (1.7)
Chronic condition, yes, *n* (%)	172 (22.0)	71 (19.7)	35 (20.1)	49 (25.9)	17 (28.3)
COVID vaccination, *n* (%)^a^					
No	327 (41.7)	182 (50.6)	107 (60.8)	23 (12.2)	15 (25.0)
Yes	147 (18.7)	25 (6.9)	37 (21.0)	66 (34.9)	19 (31.7)
Unknown	31 (3.9)	5 (1.4)	7 (4.0)	10 (5.3)	9 (15.0)
Missing data	280 (35.7)	148 (41.1)	25 (14.2)	90 (47.6)	17 (28.3)
Variant time phases, *n* (%)					
Wild type (before April 18, 2021)	206 (26.2)	106 (29.4)	14 (8.0)	76 (40.2)	10 (16.7)
Alpha (April 18–June 26, 2021)	167 (21.3)	88 (24.4)	20 (11.4)	45 (23.8)	14 (23.3)
Gamma	1 (0.1)	0 (0)	0 (0)	0 (0)	1 (1.7)
Delta (June 27, 2021–December 11, 2021)	254 (32.4)	132 (36.7)	64 (36.4)	43 (22.8)	15 (25.0)
Omicron (Dec 12, 2021–present day)	157 (20.0)	34 (9.4)	78 (44.3)	25 (13.2)	20 (33.3)
Variants^b^					
Wild type	24/236 (10.2)	^b^	14/176 (8.6)	^b^	10/60 (16.7)
Alpha	34/236 (14.4)	^b^	20/176 (11.4)	^b^	14/60 (23.3)
Gamma	1/236 (0.4)	^b^	0/176 (0)	^b^	1/60 (1.7)
Delta	79/236 (33.5)	^b^	64/176 (36.4)	^b^	15/60 (25.0)
Omicron	98/236 (41.5)	^b^	78/176 (44.3)	^b^	20/60 (33.3)
Admitted at index ED visit, *n* (%)	106 (13.5)	38 (10.6)	20 (11.4)	40 (21.2)	8 (13.3)

^a^ COVID vaccination status data collection implemented 11 June 2021, 10 months after the study start.

^b^ Applicable to children who tested positive for SARS-CoV-2.

### Construct validity

EFA of the PASC index in both age groups and time points identified two factors. Among the children 6 to <12 years old, at 6 months, factor 1 was primarily associated with physical symptoms, while factor 2 was associated with psychological/anxiety or cognitive symptoms (e.g., trouble with memory or focusing). At the 12-month follow-up, factor 1 remained associated with physical symptoms, while factor 2 was linked to fearfulness and memory problems (Table 2). The two factors explained 38.5% and 31.5% of the total variance at 6 and 12 months, respectively. For children aged 12 to <18 years, at 12 months, factor 1 included physical symptoms, whereas factor 2 reflected fatigue-related symptoms. These factors explained 39.7% of the total variance**.**

**Table 2:** Pattern matrix and factor loadings from exploratory factor analysis of post-acute sequelae of COVID-19 index including only SARS-CoV-2 positive participants. Factor analysis not performed for 12- to <18-year-old group at 6-month follow-up due to an insufficient number of participants.

SARS-CoV-2 positive participants aged 6 to <12 years				
Items	6-month follow-up		12-month follow-up	
	Factor 1	Factor 2	Factor 1	Factor 2
Nausea or vomiting	0.89	−0.10	0.51	0.40
Feeling lightheaded or dizzy	0.84	−0.12	0.20	0.28
Stomach pain	0.65	0.22	0.52	0.04
Headache	0.53	0.19	0.69	−0.08
Trouble sleeping	0.30	0.44	0.45	0.28
Fear about specific things	0.21	0.55	−0.19	0.75
Refusing to go to school	0.18	0.07	0.58	0.002
Trouble with memory or focusing	0.11	0.45	0.19	0.42
Itchy skin or skin rash	−0.04	0.04	−0.01	0.16
Back or neck pain	−0.07	0.73	0.49	−0.10
Percent of Variance	27.9%	10.7%	23.7%	7.8%
SARS-CoV-2 positive participants aged 12 to <18 years at 12-month follow-up				
	Factor 1		Factor 2	
Feeling lightheaded or dizzy	0.82		−0.02	
Headache	0.80		−0.05	
Back or neck pain	0.45		0.30	
Trouble with memory or focusing	0.27		0.53	
Daytime low energy	0.24		0.63	
Body, muscle, or joint pain	0.06		0.21	
Tired after walking	−0.08		0.59	
Change or loss of smell or taste	−0.20		0.45	
Percent of Variance	27.6%		12.1%	

### Concurrent validity

An inverse correlation, i.e., higher PASC scores and lower PedsQL^™^ scores, occurred at all time points within all age groups (Supplementary Table S5 and Figure S1). For children aged 6 to <12 years, the correlations did not differ between SARS-CoV-2 positive and negative groups, ranging from −0.71 (positives at 12 months) to −0.74 (negatives at 12 months). For participants aged 12 to <18 years, the inverse correlation was greater among SARS-CoV-2 negative participants at 6 months (difference: 0.36; 95% CI: 0.28, 0.85), but the opposite (i.e., stronger among SARS-CoV-2 positives) was detected at 12 months (difference: −0.19; 95% CI: −0.58, 0.03).

Correlation analysis between PASC index scores and overall health status revealed inverse correlations being strongest among SARS-CoV-2 positive participants aged 12 to <18 years at the 6-month follow-up (−0.59; 95% CI: −0.79, −0.28) and weakest among SARS-CoV-2 negative participants aged 6 to <12 years at the 6-month follow-up (−0.34; 95% CI: −0.47, −0.18) (Supplementary Table S6 and Figure S2). Overall, the strength of correlations did not differ between SARS-CoV-2 positive and negative groups at either time points or age groups.

### Reliability

Internal reliability was greatest among SARS-CoV-2 positive children aged 6 to <12 years at 6 months (*α* = 0.67; 95% CI: 0.64, 0.69) suggesting moderate internal consistency. It was lowest among participants aged 12 to <18 years at 6 months (*α* = 0.49; 95% CI: 0.45, 0.53) reflecting low internal consistency (Supplementary Table S7). In the generalizability analysis, the largest variance was attributable to changes in PASC scores across the two time points, accounting for 70.8% and 62.8% of the total variance, among the younger and older age cohorts, respectively. The variance attributed to individual subjects was 15.1% and 36.8%, respectively. For children aged 6 to <12 years, adding five repeated assessments would increase the G coefficient to 0.6, while adding one additional assessment yields a similar improvement for the 12- to <18-year-old group (Supplementary Table S8).

### Association with SARS-CoV-2 infection

**Figure 2:**
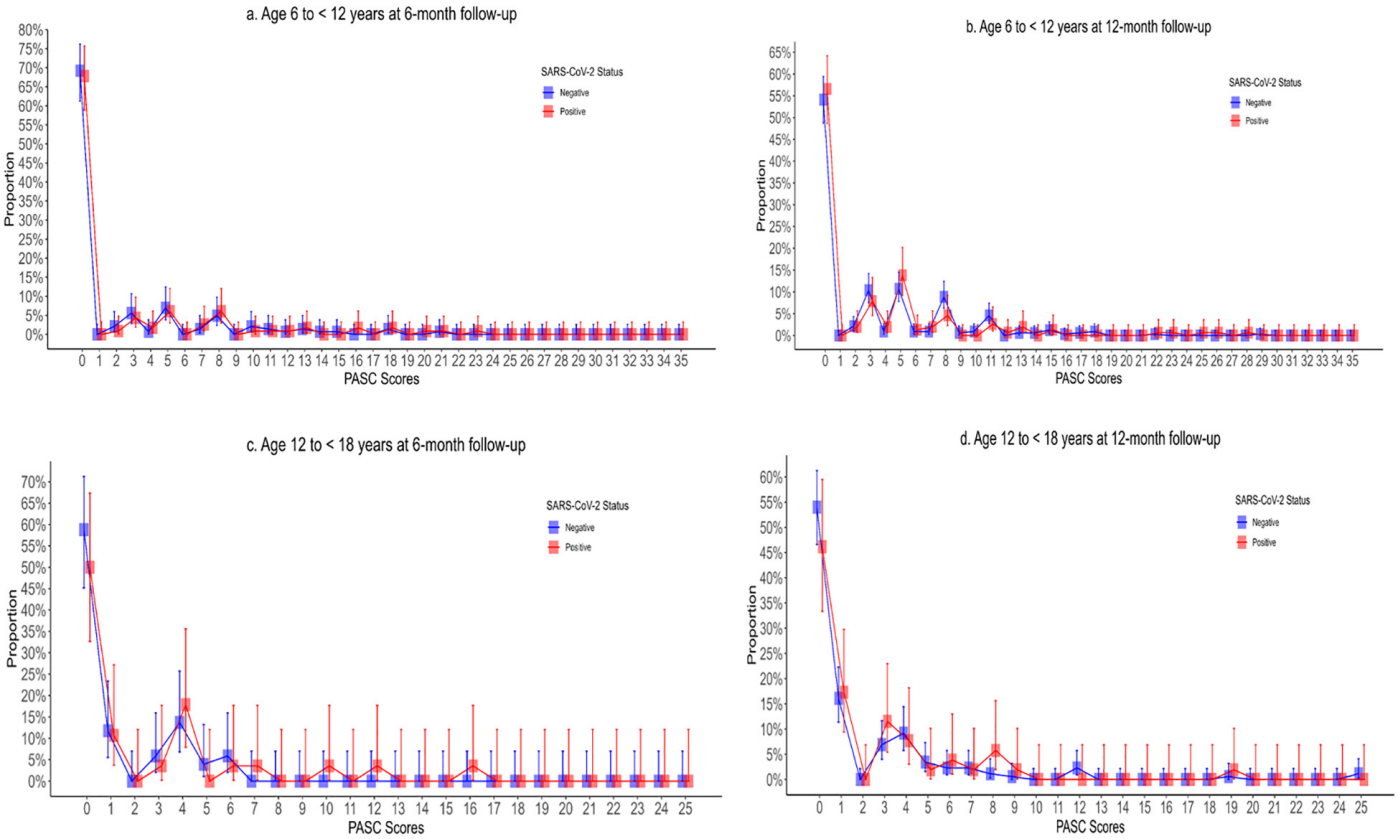
Post-acute sequelae of COVID-19 index stratified by SARS-CoV-2 test status, age, and follow-up time point with Panel **A** depicting children aged 6–12 years at 6-month follow-up; **(B)** depicting children aged 6–12 years at 12-month follow-up; **(C)** depicting children aged 12–18 years at 6-month follow-up; and **(D)** depicting 12–18 years at 12-month follow-up.

The individual symptoms reported by participants, stratified by age group, survey time point, and SARS-CoV-2 status, are reported in Supplementary Table S9 and depicted in Supplementary Figure S3. PASC scores were right skewed with means greater than medians in all age groups and time points (Table 3, Figure 2). At 6 months, 25.2% (29/115) and 21.7% (31/143) of 6- to <12-year-old SARS-CoV-2 positive and negative participants, respectively, had PASC scores exceeding the 5.5-point threshold (difference = 3.5%; 95% CI of the difference: −6.7%, 14.1%). These proportions increased to 27.0% (41/152) and 31.5% (103/327), respectively, at 12 months (difference = −4.5%; 95% CI of the difference: −12.8%, 4.4%). Among 12- to <18-year-olds, 17.9% (5/28) and 9.8% (5/51) of SARS-CoV-2 positive and negative participants had PASC scores exceeding the threshold at 6 months (difference = 8.1%; 95% CI of the difference: −6.9%, 26.6%). Among these children, at 12 months, the difference was 3.5% (95% CI of the difference: −6.4%, 16.7%). GEE analysis revealed no difference in PASC index categorization based on SARS-CoV-2 test status in either age groups or time points. The presence of a chronic pre-existing condition was however associated with a PASC score exceeding the threshold in children aged 6 to <12 years of age at the 12-month follow-up (aOR: 2.64; 95% CI: 1.82, 3.84). In our sensitivity analysis, we did find that at 6 months, 17.9% (5/28) and 2.0% (1/51) of 6- to <12-year-old SARS-CoV-2 positive and negative participants, respectively, had PASC scores exceeding the 5.5-point threshold (difference = 15.9%; 95% CI of the difference: 1.0%, 31.4%).

**Table 3:** Characteristics of the post-acute sequelae of COVID-19 index, stratified by age group, SARS-CoV-2 infection status, and follow-up time point.

Age range	SARS-CoV-2 positive			SARS-CoV-2 negative			Median difference^a^	*P*-value^b^
	*n*	Mean (SD)	Median (IQR)	*n*	Mean (SD)	Median (IQR)		
6-month follow-up								
Age 6 to <12 years	115	3.0 (5.4)	0 (0, 5.5)	143	2.4 (4.4)	0 (0, 3.0)	0 (0, 0)	0.65
Age 12 to <18 years	28	2.9 (4.3)	0.8 (0, 4.4)	51	1.5 (2.2)	0.8 (0, 3.5)	0 (0, 1)	0.26
12-month follow-up								
Age 6 to <12 years	152	3.4 (5.5)	0 (0, 5.5)	327	3.4 (4.7)	0 (0, 5.5)	0 (0, 0)	0.56
Age 12 to <18 years	52	2.4 (3.7)	1.0 (0, 4.3)	174	2.1 (3.9)	1.0 (0, 3.5)	0 (0, 0)	0.37
	Index score cut point	Total study cohort [exceed cut point/total, (%)]	SARS-CoV-2 positive		SARS-CoV-2 negative		Odds ratio (95% CI)^c^	
6-month follow-up								
Age 6 to <12 years	≥5.5	60/258 (23.3)	29/115 (25.2)		31/143 (21.7)		1.24 (0.63, 2.47)	
Age 12 to <18 years	≥5.0	10/79 (12.7)	5/28 (17.9)		5/51 (9.8)		2.19 (0.46, 10.4)	
12-month follow-up								
Age 6 to <12 years	≥5.5	144/479 (30.1)	41/152 (27.0)		103/327 (31.5)		0.81 (0.47, 1.38)	
Age 12 to <18 years	≥5.0	33/226 (14.6)	9/52 (17.3)		24/174 (13.8)		1.31 (0.50, 3.45)	

^a^ Median difference using independent-samples Hodges–Lehman test.

^b^ *P* values obtained from Mann–Whitney *U* tests and were unadjusted.

^c^ Odds ratios were obtained from logistic regression fitted in a generalized estimating equation (GEE) model with the binary outcome being the PASC index score exceeding the cut point. The model was adjusted for clustering by study sites. Separate GEE models were used for subgroups defined by age (6 to <12 years and 12 to <18 years) and time point (6 and 12 months).

## Discussion

In this study, factor analysis consistently identified two key factors, one of which was physical symptoms in all groups while the other represented neuropsychological symptoms (e.g., psychological, anxiety, cognition, fear, memory, and fatigue). There was a strong inverse correlation between the PASC index and other measures of QoL. The PASC index had low to moderate internal consistency with the largest amount of variance attributed to changes in scores across time points. SARS-CoV-2 positive children were not more likely to meet the proposed PASC index threshold scores than SARS-CoV-2 negative children. This finding was consistent across age groups (i.e., 6 to <12 years and 12 to <18 years) and time points (i.e., 6- and 12-month follow-up). These findings suggest the index captures non-specific symptoms that correlate with general well-being but that it does not sufficiently discriminate between those who have PASC from those who have a reduced QoL due to other illnesses.

In the PASC index derivation study, the research tool was developed by selecting symptoms most associated with a history of SARS-CoV-2 infection (29). The authors suggested that although the index could be used for research, they cautioned that it was not intended for use in clinical practice as children may have PASC without meeting the index threshold. Our findings confirm the authors' statement in that regard, because we found, in keeping with others (30), that there is considerable overlap in symptoms between SARS-CoV-2 positive and negative children. This finding is not surprising as prediction models generally perform poorer in new patients than in the derivation population due to overfitting (31), and as such, models should not be recommended for clinical use before external validity is established (32).

The finding that the PASC index had poor discriminatory ability could be due to limitations in the derivation cohort, in our cohort, or more likely due to the non-specific nature of symptoms. Our study builds on evidence regarding the high prevalence of non-specific symptoms in children, irrespective of SARS-CoV-2 infection history and vaccination status (30). Recurrent abdominal pain, defined by ≥3 episodes of pain occurring over ≥3 months that affect daily activities (33), which has a prevalence of 14% (6), results in a score of 5 points among 6- to <12-year-olds. Among teenagers, daytime tiredness, sleepiness, or low energy results in a score of 3.5 points; however, excessive daytime sleepiness affects 20%–35% of adolescents (34). Given the frequency of these symptoms in the population, it is not surprising that the PASC index failed to identify those likely to have PASC from those likely to have an alternate etiology for their symptoms, based on evidence of SARS-CoV-2 infection. This finding aligns with prior attempts to quantify PASC prevalence using control groups, which have reported similar proportions between test-positive and test-negative children (8, 15, 17, 35–37).

Chronic medical syndromes can occur after a variety of acute infections and are often characterized by an unexplained failure to recover from acute infection despite objective biomarker studies that are generally unremarkable and a pathogen that is rarely detectable using common methods (38). Indeed, PASC shares many similarities with such illnesses, being characterized by core symptoms of exertional intolerance, fatigue, neurocognitive impairments, and other non-specific symptoms. Ideally, a biomarker test could serve as the gold standard to identify which individuals have a post-acute infection syndrome and which pathogen has triggered their symptoms. That test could then serve as the reference to which clinical scores are evaluated. As it relates to PASC, a laboratory test that accurately distinguishes individuals with PASC from those without PASC would be useful in its diagnosis, prognosis, prevention, and treatment. Unfortunately, in a recent cohort study of >10,000 participants with and without prior SARS-CoV-2 infection, there was no evidence that any routine clinical laboratory tests are a reliable biomarker of prior infection or PASC (14).

It is unlikely that clinical scores will be able to accurately identify children as having PASC from other chronic diseases such as myalgic encephalomyelitis or chronic fatigue syndrome due to the overlap in symptoms (39). Thus, a focus on biomarkers is likely going to be required. Proteomic studies in adults with PASC have described evidence of thromboinflammation, persistent immune activation, and dysregulation (40). More recently, similar findings have been reported in children, with PASC being characterized by an increase in the expression of proinflammatory and pro-angiogenetic chemokines (41). When a machine learning model based on proteomic profiles was employed, PASC was diagnosed with an accuracy of 93%. Such approaches are likely going to be needed to address the PASC diagnostic challenge.

Our study has important limitations. We relied on symptom reporting by the caregivers of study participants, and thus reporting and recall bias are possible. While young children may not verbalize their symptoms as often or as clearly to caregivers, adolescents may perceive their symptoms differently from caregivers. Thus, there is the possibility that symptoms were underreported by participants to their caregivers. The lack of a gold standard biomarker for PASC limits the ability to validate the index against an objective measure (i.e., cannot calculate sensitivity or specificity). Importantly, our questions, although similar, were not identical to those used in the derivation study (Supplementary Table S3) (9). While the derivation study focused on the presence of symptoms that lasted for longer than 4 weeks, in our study, we enquired about symptoms that had been present in the preceding 7 days.

Our target sample size was not achieved among the 12- to <18-year-old group at 6 months; thus, we cannot rule out the possibility of a type 2 error. However, in the other three strata, there was no difference between groups in the primary outcome. Antibody testing was not performed; therefore, we cannot exclude the possibility that some participants in the negative group had been infected by SARS-CoV-2 prior to their index ED visit or that they acquired SARS-CoV-2 infection over the subsequent 12 months and thus were misclassified. Additionally, we cannot exclude the possibility that uninfected participants had another illness that triggered the development of postinfectious symptoms (e.g., post-viral fatigue) that overlap with PASC (42). As our enrolment period did not overlap with that of the RECOVER initiative, we likely had different variants infecting participants. Since different variants can lead to different acute symptoms (10), they may also lead to different PASC symptomatology.

## Conclusion

In this external evaluation study, while the scores correlate well with other measures of QoL, the PASC index did not differentiate between children who did and those who did not have SARS-CoV-2 infection. As such, clinical use of scores such as the PASC index as a diagnostic tool should be deferred until a validated tool is available. It is likely that the prevalence in the general population of many of the symptoms included in the index precludes its use to identify children with PASC. These findings underscore the need for a biomarker to identify the presence of PASC in children.

## Data Availability

The raw data supporting the conclusions of this article will be made available by the authors, in accordance with local privacy legislation, to individuals who have obtained the appropriate ethical approvals, and following the execution of the appropriate data sharing agreements.

## Members of the Pediatric Emergency Research Canada (PERC) COVID Study Group

Dr. Samina Ali, Dr. Bruce Wright, Dr. Henry Li, Tyrus Crawford, Andrea Eaton.

## References

[1] World Health Organization. A clinical case definition for Post-COVID-19 condition in children and adolescents by experts concensus (2023). Available online at: https://www.who.int/publications/i/item/WHO-2019-nCoV-Post-COVID-19-condition-CA-Clinical-case-definition-2023-1 (Accessed August 14, 2024).

[2] Centers for Disease Control and Prevention. Nearly one in five American adults who have had COVID-19 still have “long COVID” (2022). Available online at: https://www.cdc.gov/nchs/pressroom/nchs_press_releases/2022/20220622.htm#print (Accessed August 17, 2024).

[3] Rao S Lee GM Razzaghi H Lorman V Mejias A Pajor NM Clinical features and burden of postacute sequelae of SARS-CoV-2 infection in children and adolescents. JAMA Pediatr. (2022) 176(10):1000–9. 10.1001/jamapediatrics.2022.2800 35994282 PMC9396470

[4] Goldman RD. Long COVID in children. Can Fam Physician. (2022) 68(4):263–5. 10.46747/cfp.6804263 35418390 PMC9007126

[5] Onofri A Pensato U Rosignoli C Wells-Gatnik W Stanyer E Ornello R Primary headache epidemiology in children and adolescents: a systematic review and meta-analysis. J Headache Pain. (2023) 24(1):8. 10.1186/s10194-023-01541-0 36782182 PMC9926688

[6] Korterink JJ Diederen K Benninga MA Tabbers MM. Epidemiology of pediatric functional abdominal pain disorders: a meta-analysis. PLoS One. (2015) 10(5):e0126982. 10.1371/journal.pone.0126982 25992621 PMC4439136

[7] Dun-Dery F Xie J Winston K Burstein B Gravel J Emsley J Post-COVID-19 condition in children 6 and 12 months after infection. JAMA Network Open. (2023) 6(12):e2349613. 10.1001/jamanetworkopen.2023.49613 38153737 PMC10755606

[8] Stephenson T Pinto Pereira SM Shafran R de Stavola BL Rojas N McOwat K Physical and mental health 3 months after SARS-CoV-2 infection (long COVID) among adolescents in England (CLoCk): a national matched cohort study. Lancet Child Adolesc Health. (2022) 6(4):230–9. 10.1016/S2352-4642(22)00022-0 35143770 PMC8820961

[9] Gross RS Thaweethai T Kleinman LC Snowden JN Rosenzweig EB Milner JD Characterizing long COVID in children and adolescents. JAMA. (2024) 332(14):1174–88. 10.1001/jama.2024.12747 39196964 PMC11339705

[10] Sumner MW Xie J Zemek R Winston K Freire G Burstein B Comparison of symptoms associated with SARS-CoV-2 variants among children in Canada. JAMA Netw Open. (2023) 6(3):e232328. 10.1001/jamanetworkopen.2023.2328 36892839 PMC9999248

[11] Bialy L Plint A Zemek R Johnson D Klassen T Osmond M Pediatric emergency research Canada: origins and evolution. Pediatr Emerg Care. (2018) 34(2):138–44. 10.1097/PEC.0000000000001360 29189589

[12] Bossuyt PM Reitsma JB Bruns DE Gatsonis CA Glasziou PP Irwig L STARD 2015: an updated list of essential items for reporting diagnostic accuracy studies. Br Med J. (2015) 277:h5527. 10.1136/bmj.h5527 PMC4623764 26511519

[13] Peiris S Izcovich A Ordunez P Luciani S Martinez C Aldighieri S Challenges to delivering evidence-based management for long COVID. BMJ Evid Based Med. (2023) 28(5):295–8. 10.1136/bmjebm-2023-112311 37491142 PMC10579509

[14] Erlandson KM Geng LN Selvaggi CA Thaweethai T Chen P Erdmann NB Differentiation of prior SARS-CoV-2 infection and postacute sequelae by standard clinical laboratory measurements in the RECOVER cohort. Ann Intern Med. (2024) 177(9):1209–21. 10.7326/M24-0737 39133923 PMC11408082

[15] Funk AL Kuppermann N Florin TA Tancredi DJ Xie J Kim K Post-COVID-19 conditions among children 90 days after SARS-CoV-2 infection. JAMA Netw Open. (2022) 5(7):e2223253. 10.1001/jamanetworkopen.2022.23253 35867061 PMC9308058

[16] Jacobs MM Evans E Ellis C. Racial, ethnic, and sex disparities in the incidence and cognitive symptomology of long COVID-19. J Natl Med Assoc. (2023) 115(2):233–43. 10.1016/j.jnma.2023.01.016 36792456 PMC9923441

[17] Funk AL Florin TA Kuppermann N Tancredi DJ Xie J Kim K Outcomes of SARS-CoV-2-positive youths tested in emergency departments: the global PERN-COVID-19 study. JAMA Netw Open. (2022) 5(1):e2142322. 10.1001/jamanetworkopen.2021.42322 35015063 PMC8753506

[18] Varni JW Seid M Rode CA. The PedsQL: measurement model for the pediatric quality of life inventory. Med Care. (1999) 37(2):126–39. 10.1097/00005650-199902000-00003 10024117

[19] Varni JW Burwinkle TM Seid M Skarr D. The PedsQL 4.0 as a pediatric population health measure: feasibility, reliability, and validity. Ambul Pediatr. (2003) 3(6):329–41. 10.1367/1539-4409(2003)003<0329:TPAAPP>2.0.CO;2 14616041

[20] Varni WJ. Scaling and scoring for the acute and standard versions of the pediatric quality of life inventory™ (PedsQL). Lyon France Mapi Res Trust. (2023) 21(3):1–91. Available online at: https://www.pedsql.org/PedsQL-Scoring.pdf (Accessed September, 3, 2024).

[21] Adams G Gulliford MC Ukoumunne OC Eldridge S Chinn S Campbell MJ. Patterns of intra-cluster correlation from primary care research to inform study design and analysis. J Clin Epidemiol. (2004) 57(8):785–94. 10.1016/j.jclinepi.2003.12.013 15485730

[22] Agresti A Caffo B. Simple and effective confidence intervals for proportions and differences of proportions result from adding two successes and two failures. Am Stat. (2000) 54(4):280–8. 10.1080/00031305.2000.10474560

[23] Kline P. An Easy Guide to Factor Analysis. 1st ed. London: Routledge (2014).

[24] Mukaka MM. Statistics corner: a guide to appropriate use of correlation coefficient in medical research. Malawi Med J. (2012) 24(3):69–71. 23638278 PMC3576830

[25] Ponterotto JG Ruckdeschel DE. An overview of coefficient alpha and a reliability matrix for estimating adequacy of internal consistency coefficients with psychological research measures. Percept Mot Skills. (2007) 105(3 Pt 1):997–1014. 10.2466/pms.105.3.997-1014 18229554

[26] Streiner DL Norman GR Cairney J. Health Measurement Scales: A Practical Guide to Their Development and use. 5th ed. United Kingdom: Oxford University Press (2014). 10.1093/med/9780199685219.001.0001

[27] Brennan RL. (Mis) conception about generalizability theory. Educ Meas Issues Pract. (2000) 19(1):5–10. 10.1111/j.1745-3992.2000.tb00017.x

[28] Brennan RL. Performance assessments from the perspective of generalizability theory. Appl Psychol Meas. (2000) 24(4):339–53. 10.1177/01466210022031796

[29] Gross RS Thaweethai T Rosenzweig EB Chan J Chibnik LB Cicek MS Researching COVID to enhance recovery (RECOVER) pediatric study protocol: rationale, objectives and design. PLoS One. (2024) 19(5):e0285635. 10.1371/journal.pone.0285635 38713673 PMC11075869

[30] Stephenson T Pinto Pereira SM Nugawela MD Dalrymple E Harnden A Whittaker E A 24-month national cohort study examining long-term effects of COVID-19 in children and young people. Commun Med (Lond). (2024) 4(1):255. 10.1038/s43856-024-00657-x 39633013 PMC11618575

[31] Ramspek CL Jager KJ Dekker FW Zoccali C van Diepen M. External validation of prognostic models: what, why, how, when and where? Clin Kidney J. (2021) 14(1):49–58. 10.1093/ckj/sfaa188 33564405 PMC7857818

[32] Moons KG Altman DG Reitsma JB Ioannidis JPA Macaskill P Steyerberg EW Transparent reporting of a multivariable prediction model for individual prognosis or diagnosis (TRIPOD): explanation and elaboration. Ann Intern Med. (2015) 162(1):W1–73. 10.7326/M14-0698 25560730

[33] Apley J Naish N. Recurrent abdominal pains: a field survey of 1,000 school children. Arch Dis Child. (1958) 33(168):165–70. 10.1136/adc.33.168.165 13534750 PMC2012205

[34] Findlay SM. The tired teen: a review of the assessment and management of the adolescent with sleepiness and fatigue. Paediatr Child Health. (2008) 13(1):37–42. 10.1093/pch/13.1.37 19119351 PMC2528817

[35] Thaweethai T Jolley SE Karlson EW Levitan EB Levy B McComsey GA Development of a definition of postacute sequelae of SARS-CoV-2 infection. JAMA. (2023) 329(22):1934–46. 10.1001/jama.2023.8823 37278994 PMC10214179

[36] Wang C Ramasamy A Verduzco-Gutierrez M Brode WM Melamed E. Acute and post-acute sequelae of SARS-CoV-2 infection: a review of risk factors and social determinants. Virol J. (2023) 20(1):124. 10.1186/s12985-023-02061-8 37328773 PMC10276420

[37] Dun-Dery F Xie J Winston K Burstein B Emsley J Sabhaney V No association between SARS-CoV-2 infection and quality of life 6- and 12-months after infection. Acad Pediatr. (2024) 25(1):102536. 10.1016/j.acap.2024.07.003 39004300

[38] Choutka J Jansari V Hornig M Iwasaki A. Unexplained post-acute infection syndromes. Nat Med. (2022) 28(5):911–23. 10.1038/s41591-022-01810-6 35585196

[39] Morrow AK Malone LA Kokorelis C Petracek LS Eastin EF Lobner KL Long-term COVID 19 sequelae in adolescents: the overlap with orthostatic intolerance and ME/CFS. Curr Pediatr Rep. (2022) 10(2):31–44. 10.1007/s40124-022-00261-4 35287333 PMC8906524

[40] Davis HE McCorkell L Vogel JM Topol EJ. Long COVID: major findings, mechanisms and recommendations. Nat Rev Microbiol. (2023) 21(3):133–46. 10.1038/s41579-022-00846-2 36639608 PMC9839201

[41] Buonsenso D Cotugno N Amodio D Pascucci GR Di Sante G Pighi C Distinct pro-inflammatory/pro-angiogenetic signatures distinguish children with long COVID from controls. Pediatr Res. (2025):1–8. 10.1038/s41390-025-03837-0 39849114

[42] Minotti C McKenzie C Dewandel I Bekker C Sturniolo G Doni D How does post COVID differ from other post-viral conditions in childhood and adolescence (0–20 years old)? A systematic review. EClinicalMedicine. (2024) 68:102436. 10.1016/j.eclinm.2024.102436 38333536 PMC10850405

