# Supplementary material for: Evaluation of a pediatric post-acute sequelae of SARS-CoV-2 index score
Source: Front Pediatr. 2025 Sep 10;13:1628826. doi: 10.3389/fped.2025.1628826 (PMC12459272; doi:10.3389/fped.2025.1628826)
Supplement: Supplementary file 1 [file Supplementaryfile1.docx]

**Supplemental Tables and Figures**

**Supplemental Tables**

**Supplemental Table 1.** Study Sites and Distribution of Participants Recruited, stratified by SARS-CoV-2 test status

**Supplemental Table 2.** International Severe Acute Respiratory and Emerging Infection Consortium (ISARIC) Long-COVID Pediatric survey tool.

**Supplemental Table 3.** Comparison of Post-Acute Sequelae of COVID-19 index symptoms between RECOVER survey and the current study.

**Supplemental Table 4.** Baseline demographics stratified by SARS-CoV-2 test outcome and follow-up completion status.

**Supplemental Table 5.** Correlation between Post-Acute Sequelae of COVID-19 index and PedsQL scores

**Supplemental Table 6.** Correlation between Post-Acute Sequelae of COVID-19 index with overall health status scores

**Supplemental Table 7.** Reliability evaluation using Cronbach’s alpha of the Post-Acute Sequelae of COVID-19 Index scores in SARS-CoV-2 test-positive children.

**Supplemental Table 8.** Generalizability analysis of Post-Acute Sequelae of COVID-19 scores administered to children with SARS-CoV-2 infection, at 6-month and 12-month after their index ED visit.

**Supplemental Table 9.** Presence of symptoms stratified by SARS-CoV-2 test status, age, and follow-up time point

**Supplemental Figures**

**Supplemental Figure 1.** Scatter plots for relationship between Post-Acute Sequelae of COVID-19 and PedQL™ scores; values and comparisons between SARS-CoV-2 positive and negative participants are provided in eTable 5.

**Supplemental Figure 2.** Scatter plots for relationship between Post-Acute Sequelae of COVID-19 index and overall health status scores; values and comparisons between SARS-CoV-2 positive and negative participants are provided in eTable 6**.**

**Supplemental Figure 3. (panels a, b, c, and d).** Presence of symptoms stratified by SARS-CoV-2 test status, age, and follow-up time point.

**eTable 1. Study sites and distribution of eligible participants, stratified by SARS-CoV-2 test status.**

| **Study sites** | **SARS-CoV-2 Status** | | **Sub-Total (%)** |
| --- | --- | --- | --- |
|  | **Negative** | **Positive** |  |
| Montreal Children’s Hospital | 154 | 40 | 194 (24.7%) |
| Alberta Children’s Hospital (Calgary) | 76 | 56 | 132 (16.8%) |
| BC Children’s Hospital (Vancouver) | 75 | 16 | 91 (11.6%) |
| McMaster Children’s Hospital (Hamilton) | 57 | 11 | 68 (8.7%) |
| IWK Children’s Health Centre (Halifax) | 43 | 11 | 54 (6.9%) |
| The Children’s Hospital of Winnipeg | 42 | 8 | 50 (6.4%) |
| CHU Sainte-Justine (Montreal) | 15 | 33 | 48 (6.1%) |
| Children’s Hospital of Eastern Ontario (Ottawa) | 13 | 24 | 37 (4.7%) |
| The Hospital for Sick Children (Toronto) | 12 | 23 | 35 (4.5%) |
| Jim Pattison Children’s Hospital (Saskatoon) | 31 | 3 | 34 (4.3%) |
| Centre Hospitalier de l’Université de Laval (Quebec City) | 7 | 10 | 17 (2.2%) |
| Janeway Children’s Health and Rehabilitation Centre (St. Johns) | 12 | 0 | 12 (1.5%) |
| Children’s Hospital London Health Sciences Centre | 9 | 1 | 10 (1.3%) |
| Kingston Health Sciences Centre | 3 | 0 | 3 (0.4%) |
| Total | 549 | 236 | 785 (100%) |

**eTable 2: International Severe Acute Respiratory and Emerging Infection Consortium (ISARIC) Long-COVID Pediatric survey tool.**


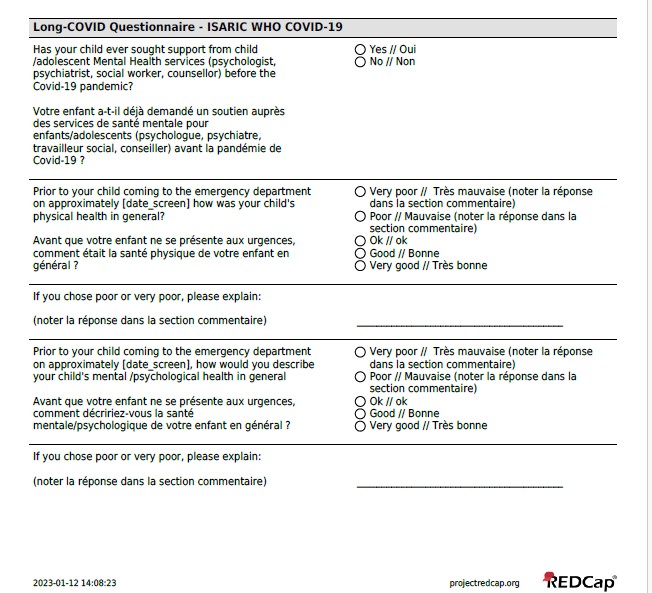


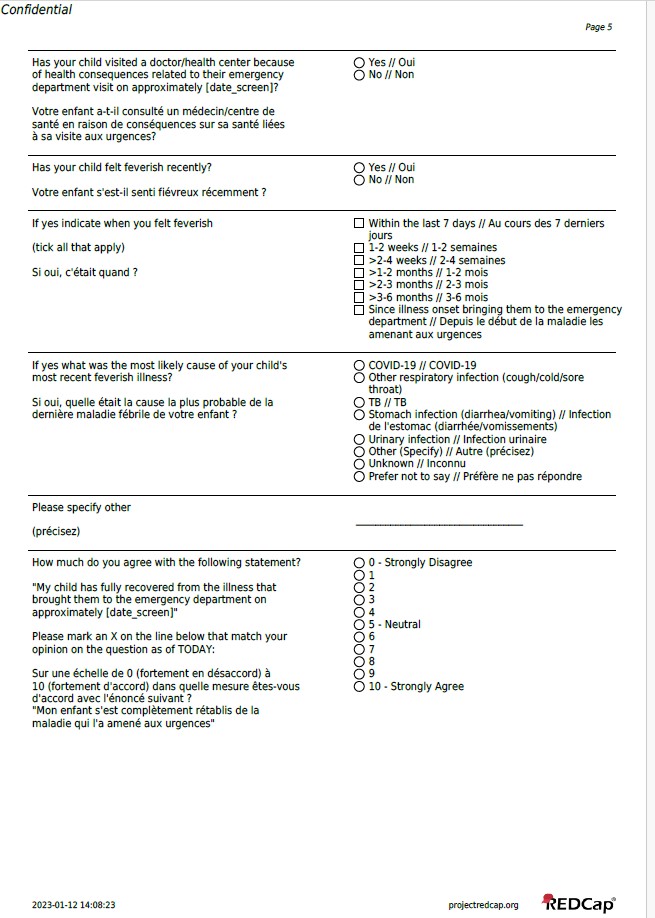


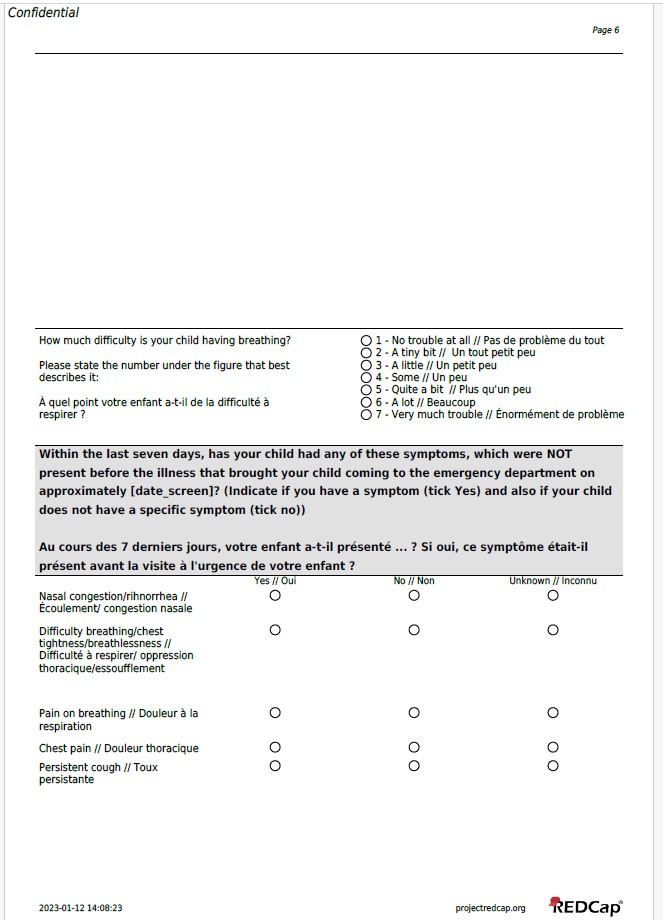


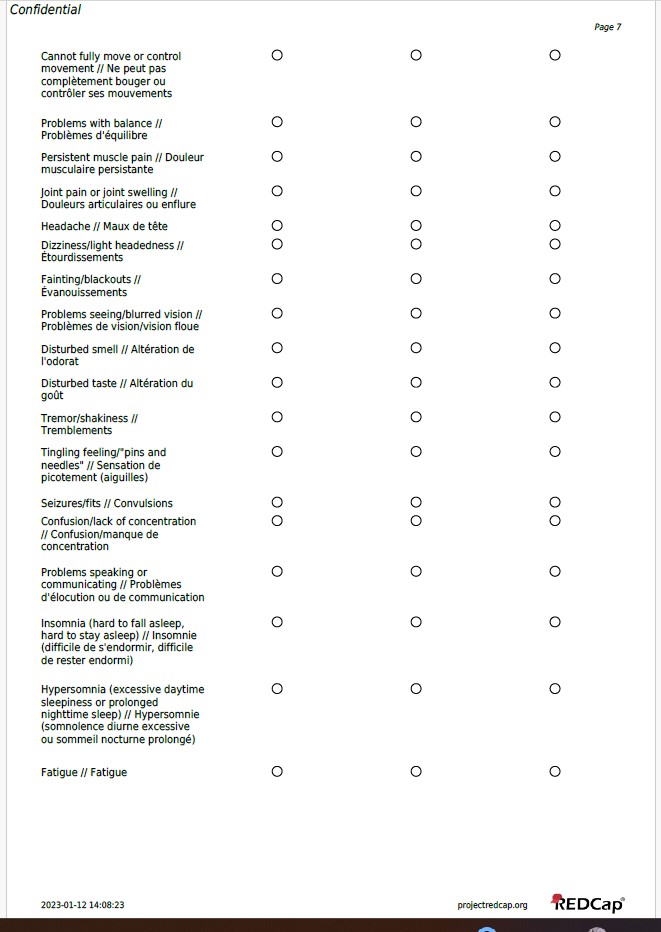


**eTable 3. Comparison of** **Post-Acute Sequelae of COVID-19 index symptoms between RECOVER survey and the current study.**

| **Symptom as they Appear**  **in the RECOVER Survey** | | **RECOVER Index Score Symptom Criteria^1^** | **Matched Question in our Survey** | **Score assigned in RECOVER Study** |
| --- | --- | --- | --- | --- |
| **Symptom** | **Symptom Description** |  |  |  |
| **Young Children (6 to < 12 years)** | | | | |
| Memory problems | Problems with remembering things (memory) | Trouble with memory or focusing | Confusion/lack of Concentration | 5.5 |
| Brain fog | Problems with focusing on things (concentration), sometimes called "brain fog" |  |  |  |
| Back pain | Pain in the back | Back or Neck pain | Persistent muscle pain | 5.0 |
| Neck pain | Pain in the neck |  |  |  |
| Stomach pain | Stomach pains/cramps | Stomach pain | Stomach/abdominal pain | 5.0 |
| Headache | Headache | Headache | Headache | 4.5 |
| Fear about specific things | Feeling a lot of fear of specific things like spiders or being up high | Fear about specific things | Feeling afraid or scared (Sometimes, Often, Almost Always)**^#^** | 3.0 |
| Refusing to go to school | Refusing to go to school | Refusing to go to school | Missing school/daycare because of not feeling well (Sometimes, Often, Almost Always) | 3.0 |
| Skin rash | Skin rash | Itchy skin or  skin rash | Skin rash | 3.0 |
| Itchy skin | Itchiness of the skin |  |  |  |
| Trouble sleeping | Trouble sleeping | Trouble sleeping | Trouble sleeping (Sometimes, Often, Almost Always) | 2.5 |
| Nausea | Nausea (feeling like you are going to throw up) | Nausea or  vomiting | Feeling nauseous, vomiting | 2.5 |
| Vomiting | Throwing up (vomiting) |  |  |  |
| Feeling dizzy | Feeling dizzy (feeling like the room is spinning) | Feeling  light-headed or dizzy | Dizziness/lightheadedness | 0.5 |
| Lightheaded | Fainting or feeling like you are going to faint (lightheaded) |  |  |  |
| **Adolescents (12 to <18 years)** | | | |  |
| Change in smell | Change in smell | Change or loss in smell or taste | Disturbed smell or taste | 12.0 |
| Loss of smell | Loss of smell |  |  |  |
| Taste changed | Change in how things taste |  |  |  |
| Muscle pain | Sore muscles or pain in the muscles | Body, muscle, or joint pain: | Joint pain or join swelling | 3.5 |
| Body pain | Body aches or pains |  |  |  |
| Joint pain | Pains in the joints (like the elbows, knees, ankles) |  |  |  |
| Sleepy | Feeling sleepy during the daytime | Daytime tiredness, sleepiness, or low energy | Hypersomnia, low energy level, fatigue | 3.5 |
| Low energy | Low energy or not feeling strong enough to do things |  |  |  |
| Tired all day long | Feeling very tired all day long |  |  |  |
| Tired after walking | Feeling very tired after walking | Tired after walking | 1. Problem with walking (Sometimes, Often, Almost Always) OR 2. Some/a lot of problems walking about (Sometimes, Often, Almost Always) OR 3. Walking more than one block (Sometimes, Often, Almost Always) | 3.0 |
| Back pain | Pain in the back | Back or neck pain | Persistent muscle pain | 1.0 |
| Neck pain | Pain in the neck |  |  |  |
| Memory problems | Problems with remembering things (memory) | Trouble with memory or focusing | Confusion/lack of Concentration | 1.0 |
| Brain fog | Problems with focusing on things (concentration), sometimes called "brain fog" |  |  |  |
| Headache | Headache | Headache | Headache | 0.5 |
| Feeling dizzy | Feeling dizzy (feeling like the room is spinning) | Feeling lightheaded or dizzy | Dizziness/lightheadedness | 0.5 |
| Lightheaded | Fainting or feeling like you are going to faint (lightheaded) |  |  |  |

#: Responses such as “Sometimes”, “Often”, and “Almost always”, are defined as positive while “Almost never”, and “Never” are defined as negative or absent

**eTable 4.** Baseline demographics stratified by SARS-CoV-2 test outcome and follow-up completion status.

1. **6-Month Follow-Up.**

|  | **SARS-CoV-2 Negative** | | | **SARS-CoV-2 Positive** | |
| --- | --- | --- | --- | --- | --- |
|  | **Lost to Follow-up** | **Completed**  **Follow-up** | **Lost to Follow-up** | | **Completed**  **Follow-up** |
|  | n=71 | n=194 | n=57 | | n=143 |
| **Age, years, median (IQR)** | 10 (8, 13) | 9 (7, 12) | 10 (8, 14) | | 9 (7, 11) |
| **Sex, male, n (%)** | 42 (59.2) | 94 (48.5) | 30 (52.6) | | 76 (53.1) |
| **Race, n (%)** |  |  |  | |  |
| Black | 9 (12.7) | 9 (4.6) | 11 (19.3) | | 14 (9.8) |
| East Asian | 3 (4.2) | 11 (5.7) | NA | | 6 (4.2) |
| Indigenous | 4 (5.6) | 6 (3.1) | 2 (3.5) | | 3 (2.1) |
| Latin American | 1 (1.4) | 13 (6.7) | 2 (3.5) | | 3 (2.1) |
| Middle Eastern | 3 (4.2) | 29 (14.9) | 6 (10.5) | | 18 (12.6) |
| Multiracial | 11 (15.5) | 30 (15.5) | 8 (14.0) | | 19 (13.3) |
| NA or unspecified | 2 (2.8) | 2 (1.0) | 1 (1.8) | | 6 (4.2) |
| South Asian | 5 (7.0) | 8 (4.1) | 3 (5.3) | | 12 (8.4) |
| Southeast Asian | 3 (4.2) | 7 (3.6) | 9 (15.8) | | 3 (2.1) |
| White | 30 (42.3) | 79 (40.7) | 15 (26.3) | | 59 (41.3) |
| **Chronic condition, n (%)** |  |  |  | |  |
| No | 52 (73.2) | 150 (77.3) | 41 (71.9) | | 110 (76.9) |
| Yes | 19 (26.8) | 44 (22.7) | 16 (28.1) | | 31 (21.7) |
| Missing data | 0 (0) | 0 (0) | 0 (0) | | 2 (1.4) |
| **COVID Vaccination, n (%)†** |  |  |  | |  |
| No | 42 (59.2) | 132 (68.0) | 32 (56.1) | | 86 (60.1) |
| Yes | 18 (25.4) | 58 (29.9) | 19 (33.3) | | 47 (32.9) |
| Unknown | 2 (2.8) | 0 (0) | 5 (8.8) | | 6 (4.2) |
| Missing data | 9 (12.7) | 4 (2.1) | 1 (1.8) | | 4 (2.8) |
| **Variant time phases, n (%)** |  |  |  | |  |
| Wild type (before April 18, 2021) | 2 (2.8) | 0 (0) | 0 (0) | | 1 (0.7) |
| Alpha (April 18-June 26, 2021) | 17 (23.9) | 23 (11.9) | 6 (10.5) | | 6 (4.2) |
| Delta (June 27, 2021-Dec 11, 2021) | 45 (63.4) | 129 (66.5) | 20 (35.1) | | 60 (42.0) |
| Omicron (Dec 12, 2021-Present day) | 7 (9.9) | 42 (21.6) | 31 (54.4) | | 75 (52.4) |
| **Variants*** |  |  |  | |  |
| Alpha | n/a | n/a | 6 (10.5) | | 6 (4.2) |
| Delta | n/a | n/a | 20 (35.1) | | 60 (42.0) |
| Omicron | n/a | n/a | 31 (54.4) | | 75 (52.4) |
| Wild type | n/a | n/a | 0 (0) | | 1 (0.7) |
| Gamma | n/a | n/a | 0 (0) | | 1 (0.7) |
| **Admitted at index ED visit, n (%)** |  |  |  | |  |
| No | 64 (90.1) | 162 (83.5) | 50 (87.7) | | 133 (93.0) |
| Yes | 7 (9.9) | 32 (16.5) | 7 (12.3) | | 10 (7.0) |

†COVID vaccination status data collection implemented June 11, 2021, 10 months after study start.

* Applicable to children who tested positive for SARS-CoV-2.

1. **12-Month Follow-Up.**

|  | **SARS-CoV-2 Negative** | | **SARS-CoV-2 Positive** | |
| --- | --- | --- | --- | --- |
|  | **Lost to Follow-up** | **Completed**  **Follow-up** | **Lost to**  **Follow-up** | **Completed**  **Follow-up** |
|  | n=248 | n=501 | n=148 | n=204 |
| **Age, years, median (IQR)** | 10 (7, 13) | 10 (7, 13) | 11 (9, 14) | 9 (7, 12) |
| **Sex, male, n (%)** | 119 (48.0) | 254 (50.7) | 84 (56.8) | 109 (53.4) |
| **Race, n (%)** |  |  |  |  |
| Black | 17 (6.9) | 19 (3.8) | 24 (16.2) | 23 (11.3) |
| East Asian | 7 (2.8) | 23 (4.6) | 1 (0.7) | 7 (3.4) |
| Indigenous | 15 (6.0) | 15 (3.0) | 8 (5.4) | 3 (1.5) |
| Latin American | 7 (2.8) | 25 (5.0) | 11 (7.4) | 8 (3.9) |
| Middle Eastern | 15 (6.0) | 44 (8.8) | 23 (15.5) | 30 (14.7) |
| Multiracial | 20 (8.1) | 71 (14.2) | 19 (12.8) | 22 (10.8) |
| NA or unspecified | 5 (2.0) | 4 (0.8) | 3 (2.0) | 9 (4.4) |
| South Asian | 9 (3.6) | 25 (5.0) | 12 (8.1) | 19 (9.3) |
| Southeast Asian | 10 (4.0) | 15 (3.0) | 11 (7.4) | 7 (3.4) |
| White | 143 (57.7) | 260 (51.9) | 36 (24.3) | 76 (37.3) |
| **Chronic condition, n (%)** |  |  |  |  |
| No | 191 (77.0) | 390 (77.8) | 109 (73.6) | 158 (77.5) |
| Yes | 57 (23.0) | 111 (22.2) | 38 (25.7) | 44 (21.6) |
| Missing data | 0 (0) | 0 (0) | 1 (0.7) | 2 (1.0) |
| **COVID Vaccination, n (%)†** |  |  |  |  |
| No | 79 (31.9) | 172 (34.3) | 59 (39.9) | 102 (50.0) |
| Yes | 28 (11.3) | 76 (15.2) | 21 (14.2) | 49 (24.0) |
| Unknown | 17 (6.9) | 15 (3.0) | 22 (14.9) | 12 (5.9) |
| Missing data | 124 (50.0) | 238 (47.5) | 46 (31.1) | 41 (20.1) |
| **Variant time phases, n (%)** |  |  |  |  |
| Wild type (before April 18, 2021) | 115 (46.4) | 182 (36.3) | 37 (25.0) | 24 (11.8) |
| Alpha (April 18-June 26, 2021) | 44 (17.7) | 128 (25.5) | 31 (20.9) | 31 (15.2) |
| Delta (June 27, 2021-Dec 11, 2021) | 77 (31.0) | 140 (27.9) | 42 (28.4) | 60 (29.4) |
| Omicron (Dec 12, 2021-Present day) | 12 (4.8) | 51 (10.2) | 34 (23.0) | 89 (43.6) |
| **Admitted at index ED visit, n (%)** |  |  |  |  |
| No | 216 (87.1) | 427 (85.2) | 135 (91.2) | 178 (87.3) |
| Yes | 32 (12.9) | 74 (14.8) | 13 (8.8) | 26 (12.7) |
| **Variants*** |  |  |  |  |
| Wild type | n/a | n/a | 37 (25.0) | 24 (11.8) |
| Alpha | n/a | n/a | 31 (20.9) | 31 (15.2) |
| Beta | n/a | n/a | 1 (0.7) | 0 (0) |
| Gamma | n/a | n/a | 3 (2.0) | 0 (0) |
| Delta | n/a | n/a | 42 (28.4) | 60 (29.4) |
| Omicron | n/a | n/a | 34 (23.0) | 89 (43.6) |

†COVID vaccination status data collection implemented June 11, 2021, 10 months after study start.

* Applicable to children who tested positive for SARS-CoV-2.

**eTable 5. Correlation between Post-Acute Sequelae of COVID-19 index and PedsQL scores.**

|  | **SARS-CoV-2 Positive** | | **SARS-CoV-2 Negative** | |  | |
| --- | --- | --- | --- | --- | --- | --- |
|  | **N** | **Pearson correlation coefficient (95%CI)** | **N** | **Pearson correlation coefficient (95%CI)** | **Difference (95%CI)** | **P-Value** |
| **6-Month Follow-Up** | | | | | | |
| Age 6 – < 12 years | 114 | -0.74 (-0.82, -0.65) | 143 | -0.72 (-0.79, -0.63) | -0.02 (-0.29, 0.20) | 0.71 |
| Age 12 – <18 years | 28 | -0.51 (-0.74, -0.17) | 51 | -0.87 (-0.93, -0.79) | 0.36 (0.28, 0.85) | 0.002 |
| **12-Month Follow-Up** | | | | | | |
| Age 6 – < 12 years | 152 | -0.71 (-0.78, -0.62) | 327 | -0.74 (-0.79, -0.69) | 0.04 (-0.12, 0.26) | 0.46 |
| Age 12 – < 18 years | 52 | -0.76 (-0.86, -0.62) | 173 | -0.58 (-0.67, -0.47) | -0.19 (-0.58, -0.03) | 0.03 |

**eTable 6. Correlation between Post-Acute Sequelae of COVID-19 with overall health status scores, stratified by SARS-CoV-2 test status, age, and follow-up time-point.**

|  | **SARS-CoV-2 Positive** | | **SARS-CoV-2 Negative** | |  | |
| --- | --- | --- | --- | --- | --- | --- |
|  | **N** | **Pearson correlation coefficient (95%CI)** | **N** | **Pearson correlation coefficient (95%CI)** | **Difference (95%CI)** | **P-Value** |
| **6-Month Follow-Up** | | | | | | |
| Age 6 – < 12 years | 115 | -0.22 (-0.37, -0.04) | 142 | -0.34 (-0.47, -0.18) | 0.12 ( -0.12, 0.36) | 0.32 |
| Age 12 – <18 years | 28 | -0.59 (-0.79, -0.28) | 51 | -0.35 (-0.57, -0.09) | -0.24 (-0.66, 0.17) | 0.21 |
| **12-Month Follow-Up** | | | | | | |
| Age 6 – < 12 years | 152 | -0.35 (-0.48, -0.21) | 324 | -0.43 (-0.51, -0.33) | 0.07 (-0.11, 0.27) | 0.39 |
| Age 12 – < 18 years | 51 | -0.54 (-0.71, -0.31) | 174 | -0.36 (-0.48, -0.22) | -0.18 ( -0.49, 0.10) | 0.18 |

**eTable 7. Reliability evaluation using Cronbach’s alpha of the Post-Acute Sequelae of COVID-19 index in SARS-CoV-2 test-positive children, grouped by age, and follow-up time point**

|  | **Cronbach's Alpha** (95%CI) | **N of Items** |
| --- | --- | --- |
| **6-Month Follow-Up** | | |
| 6 to <12 years (n=115) | 0.67 (0.64, 0.69) | 10 |
| 12 to <18 years (n=28) | 0.49 (0.45, 0.53) | 8 |
| **12-Month Follow-Up** | | |
| 6 to <12 years (n=152) | 0.65 (0.63, 0.68) | 10 |
| 12 to <18 years (n=52) | 0.53 (0.50, 0.57) | 8 |

**eTable 8. Generalizability analysis of Post-Acute Sequelae of COVID-19 index administered to children with SARS-CoV-2 infection, at 6- and 12-month post-index ED visit.**

|  | **6 to <12 Years^a^** | | **12 to <18 Years^b^** | |
| --- | --- | --- | --- | --- |
| **Source of variation** | **Estimate variance** | **%** | **Estimate variance** | **%** |
| Subject (s) | 3.39 | 15.1% | 5.47 | 36.8% |
| Repeated assessment (r) | 0.49 | 2.2% | -0.25 | 0% |
| Study site (site) | 0.07 | 0.3% | 0.07 | 0.5% |
| s × r | 15.9 | 70.8% | 9.34 | 62.8% |
| s × site | 0 | 0% | 0 | 0% |
| r × site | 2.63 | 11.7% | -1.15 | 0% |
| s × r × site, residual | 0 | 0.0% | 0 | 0% |
| **Generalizability coefficient (G)** | | | | |
| G (relative)^c^ | 0.30 | | 0.54 | |
| G (absolute)^c^ | 0.29 | | 0.54 | |
| Number of assessments needed for G approaching 0.6^d^ | 7 | | 3 | |
| G (relative) after additional repeated assessments ^d^ | 0.60 | | 0.64 | |
| G (absolute)^d^ | 0.58 | | 0.63 | |

The G coefficient should be interpreted as:

0.80 or higher: Good to excellent reliability. This indicates that a large proportion of the variance is due to true differences between subjects, and the measurement has high consistency.

0.70 to 0.79: Acceptable reliability for most purposes. The measurement is relatively stable, but there is some room for improvement.

0.60 to 0.69: Moderate or questionable reliability. It may be adequate for exploratory research, but more precision is needed for clinical or high-stakes decision-making.

Below 0.60: Low reliability. This suggests that a large proportion of the variance is due to error, and the measurement may need significant refinement (e.g., by increasing the number of items or assessments).

^a^ Included 91 children aged 6 to <12 years from 10 study sites who had PASC assessed at 6- and 12-month post index ED visit.

^b^ Included 20 children aged 12 to <18 years from 8 study sites who had PACS assessed at 6- and 12-month post index ED visit.

^c^ In the 6 to <12 year age group, the G coefficient was calculated with data available from 10 participating study sites and the measure repeated twice. In the 12 to <18-year age group, the G coefficient was calculated with data available from 8 participating study sites and number of assessments being 2.

^d^ In the 6 to <12 year age group, the G coefficient was calculated with the number of site being 1 and measure repeated 7 times. In the 12 to <18-year age group, the G coefficient was calculated with the number of sites being 1 and number of assessments being 3.

**eTable 9. Presence of symptoms stratified by SARS-CoV-2 test status, age, and follow-up time point.**

| **Aged 6 years to < 12 years** | | | | | | |
| --- | --- | --- | --- | --- | --- | --- |
|  | **6-Month Survey** | | | **12-Month Survey** | | |
|  | **Total** | **SARS-CoV-2 negative** | **SARS-CoV-2 positive** | **Total** | **SARS-CoV-2 negative** | **SARS-CoV-2 positive** |
|  | n=258 | n=143 | n=115 | N=479 | n=327 | n=152 |
| Trouble with memory or focusing, n (%) | 45 (17.4) | 24 (16.8) | 21 (18.3) | 111 (23.2) | 83 (25.4) | 28 (18.4) |
| Back or neck pain, n (%) | 9 (3.5) | 4 (2.8) | 5 (4.3) | 12 (2.5) | 8 (2.4) | 4 (2.6) |
| Stomach pain, n (%) | 11 (4.3) | 1 (0.7) | 10 (8.7) | 25 (5.2) | 11 (3.4) | 14 (9.2) |
| Headache, n (%) | 21 (8.1) | 12 (8.4) | 9 (7.8) | 32 (6.7) | 18 (5.5) | 13 (8.6) |
| Fear about specific things, n (%) | 23 (8.9) | 12 (8.4) | 11 (9.6) | 57 (11.9) | 37 (11.3) | 20 (13.2) |
| Refusing to go to school, n (%) | 28 (10.9) | 17 (11.9) | 11 (9.6) | 88 (18.4) | 69 (21.1) | 19 (12.5) |
| Itchy skin or skin rash, n (%) | 3 (1.2) | 2 (1.4) | 1 (0.9) | 14 (2.9) | 9 (2.8) | 5 (3.3) |
| Trouble sleeping, n (%) | 19 (7.4) | 11 (7.7) | 8 (7.0) | 61 (12.3) | 41 (12.5) | 20 (13.2) |
| Nausea or vomiting, n (%) | 11 (4.3) | 4 (2.8) | 7 (6.1) | 16 (3.3) | 5 (1.5) | 11 (7.2) |
| Feeling lightheaded or dizzy, n (%) | 4 (1.6) | 1 (0.7) | 3 (2.6) | 9 (1.9) | 6 (1.8) | 3 (2.0) |
| Change or loss in smell or taste, n (%) | 3 (1.2) | 1 (0.7) | 2 (1.7) | 4 (0.8) | 2 (0.6) | 2 (1.3) |
| Body, muscle, or joint pain, n (%) | 4 (1.6) | 2 (1.4) | 2 (1.7) | 13 (2.7) | 9 (2.8) | 4 (2.6) |
| Daytime low energy, n (%) | 31 (12.0) | 15 (10.5) | 16 (13.9) | 61 (12.3) | 44 (13.5) | 17 (11.2) |
| Tried after walking, n (%) | 11 (4.3) | 5 (3.5) | 6 (5.2) | 43 (9.0) | 32 (9.8) | 11 (7.2) |
| **Aged 12 years to < 18 years** | | | | | | |
|  | **6-Month Survey** | | | **12-Month Survey** | | |
|  | **Total** | **SARS-CoV-2 negative** | **SARS-CoV-2 positive** | **Total** | **SARS-CoV-2 negative** | **SARS-CoV-2 positive** |
|  | n=79 | n=51 | n=28 | n=226 | n=174 | n=52 |
| Trouble with memory or focusing, n (%) | 24 (30.4) | 13 (25.5) | 11 (39.3) | 74 (32.7) | 53 (30.5) | 21 (40.4) |
| Back or neck pain, n (%) | 3 (3.8) | 2 (3.9) | 1 (3.6) | 17 (7.5) | 13 (7.5) | 4 (7.7) |
| Stomach pain, n (%) | 8 (10.1) | 7 (13.7) | 1 (3.6) | 16 (7.1) | 10 (5.7) | 6 (11.5) |
| Headache, n (%) | 6 (7.6) | 3 (5.9) | 3 (10.7) | 27 (11.9) | 21 (12.1) | 6 (11.5) |
| Fear about specific things, n (%) | 11 (13.9) | 7 (13.7) | 4 (14.3) | 41 (18.1) | 28 (16.1) | 13 (25.0) |
| Refusing to go to school, n (%) | 17 (21.5) | 11 (22.0)* | 6 (21.4) | 58 (25.7) | 40 (23.0) | 18 (35.3)* |
| Itchy skin or skin rash, n (%) | 4 (5.1) | 1 (2.0) | 3 (10.7) | 4 (1.8) | 4 (2.3) | 0 (0) |
| Trouble sleeping, n (%) | 16 (20.3) | 11 (21.6) | 5 (17.9) | 54 (23.9) | 37 (21.3) | 17/51 (33.3) |
| Nausea or vomiting, n (%) | 7 (8.9) | 4 (7.8) | 3 (10.7) | 11 (4.9) | 9 (5.2) | 2 (3.8) |
| Feeling lightheaded or dizzy, n (%) | 2 (2.5) | 1 (2.0) | 1 (3.6) | 18 (8.0) | 13 (7.5) | 5 (9.6) |
| Change or loss in smell ot taste, n (%) | 1 (1.3) | 0 (0) | 1 (3.6) | 5 (2.2) | 4 (2.3) | 1 (1.9) |
| Body, muscle, or joint pain, n (%) | 4 (5.1) | 2 (3.9) | 2 (7.1) | 14 (6.2) | 13 (7.5) | 1 (1.9) |
| Daytime low energy, n (%) | 21 (26.6) | 13 (25.5) | 8 (28.6) | 53 (23.5) | 35 (20.1) | 18 (34.6) |
| Tired after walking, n (%) | 10 (12.7) | 3 (5.9) | 7 (25.0) | 30 (13.3) | 24 (13.8) | 6 (11.5) |

*Denominator = 50.

**eFigure 1. Scatter plots for relationship between Post-Acute Sequelae of COVID-19 and PedQL™ scores; values and comparisons between SARS-CoV-2 positive and negative participants are provided in eTable 5.**


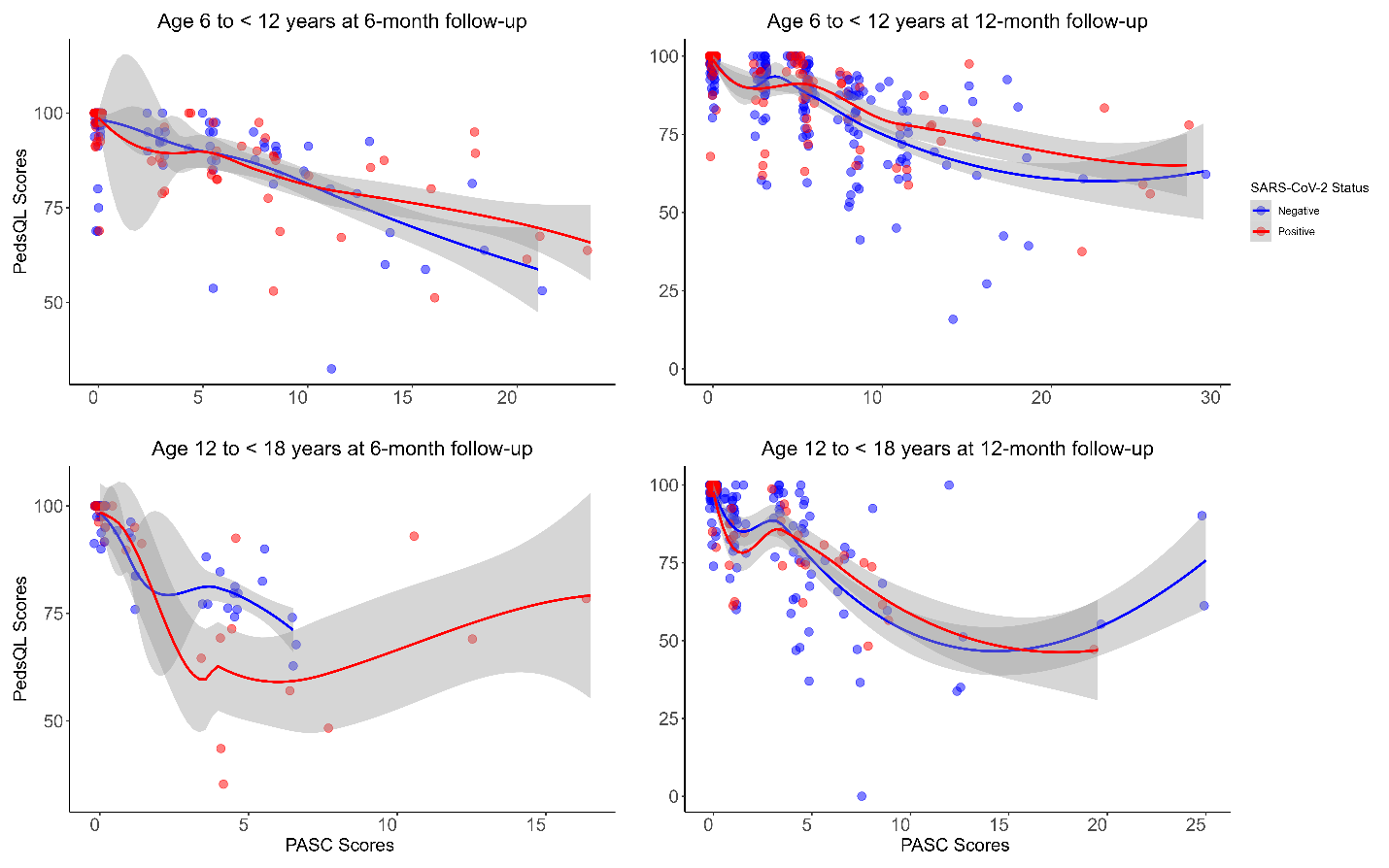


**eFigure 2. Scatter plots for relationship between Post-Acute Sequelae of COVID-19 index and overall health status scores.**


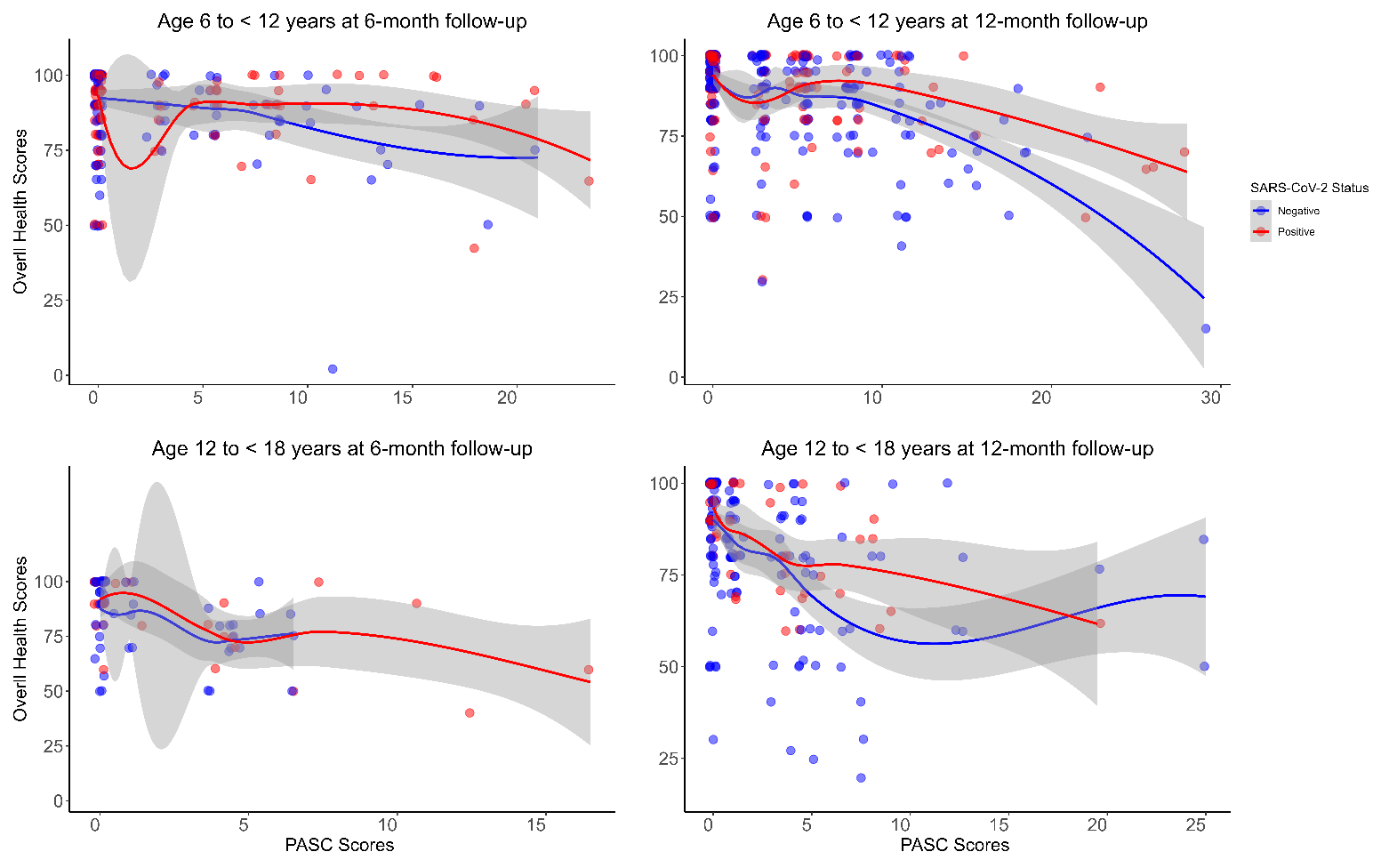


**eFigure 3 (a, b, c, and d). Presence of individual symptoms stratified by SARS-CoV-2 test status, age, and follow-up time point.**


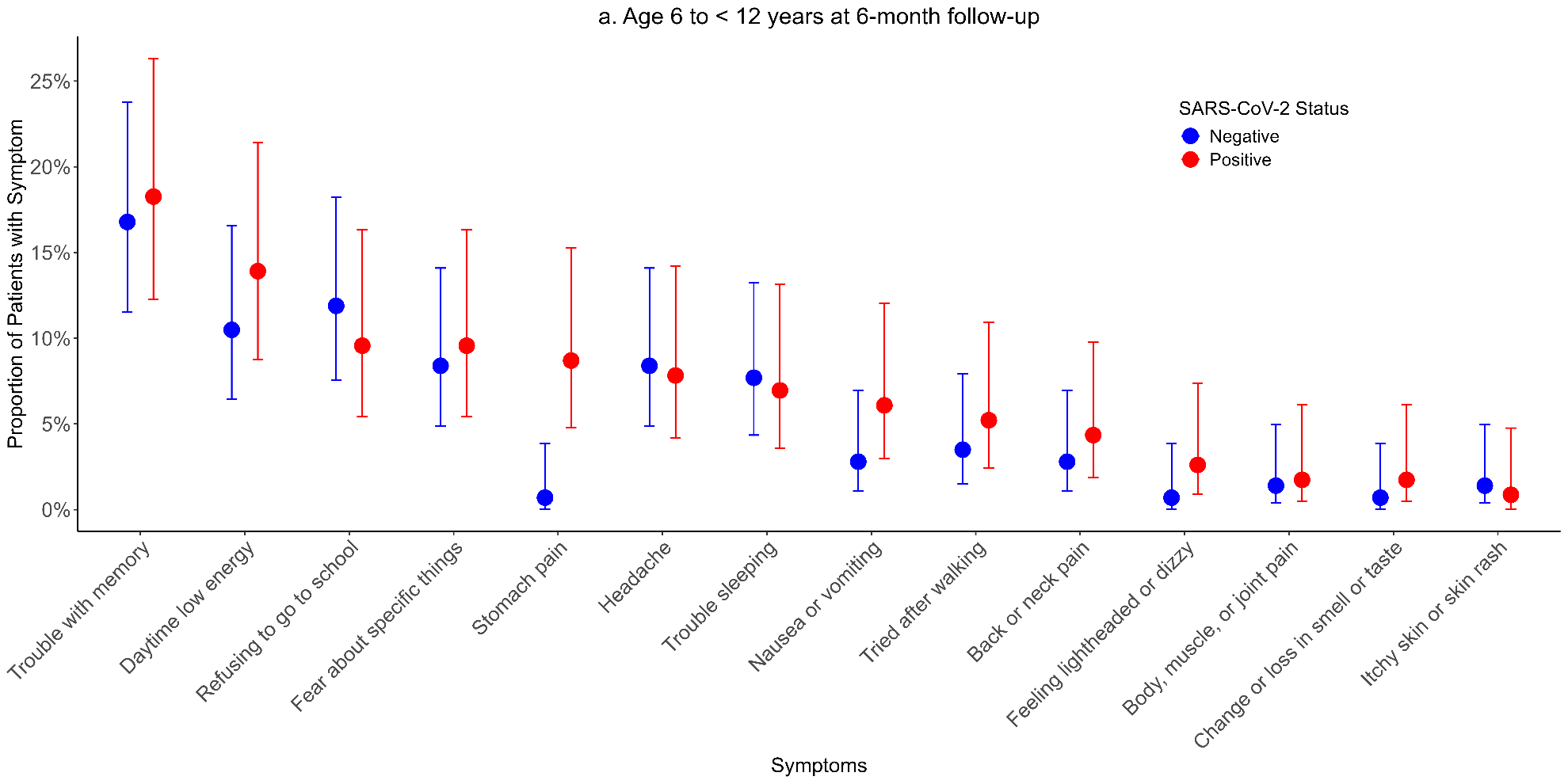


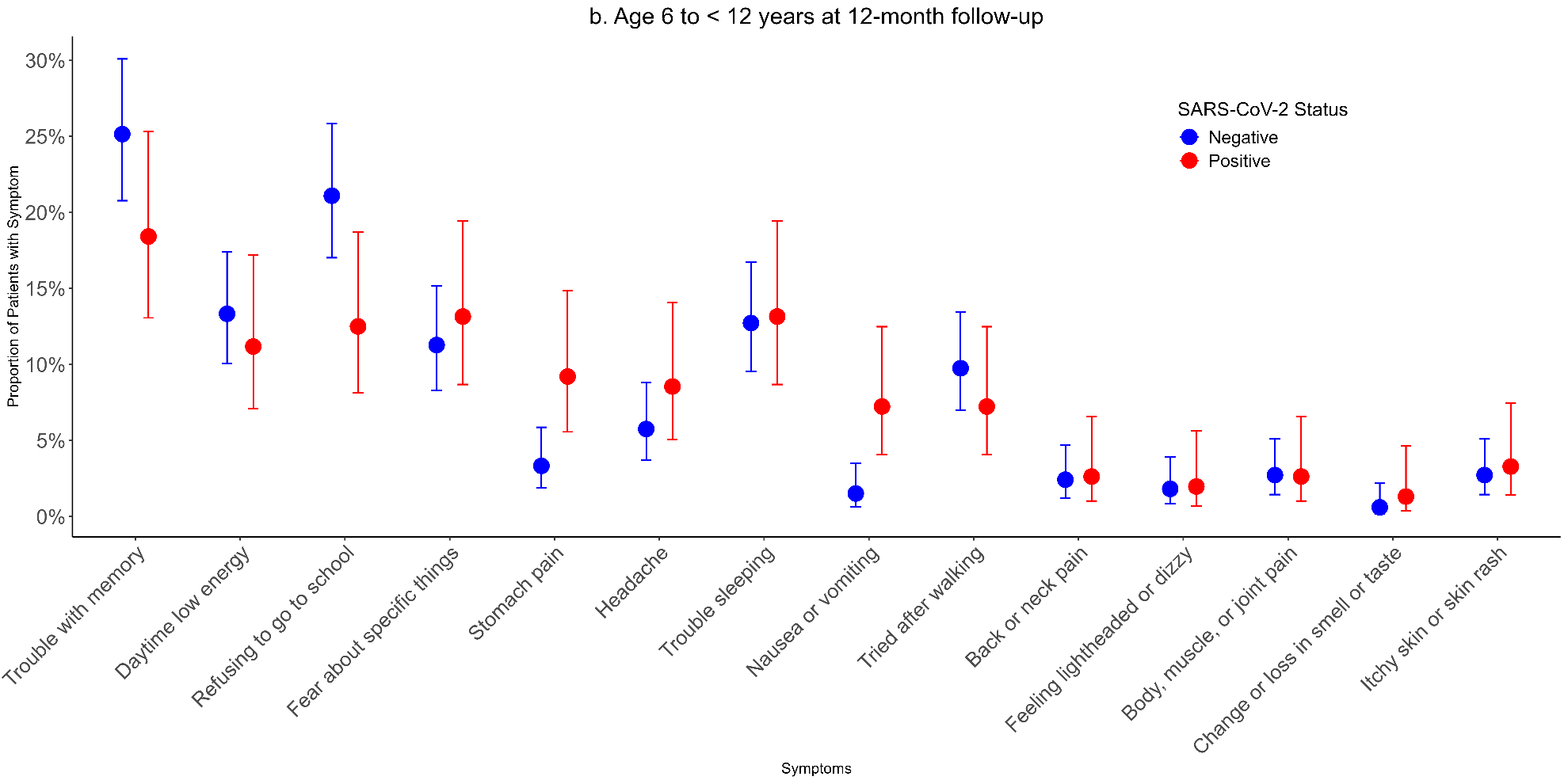


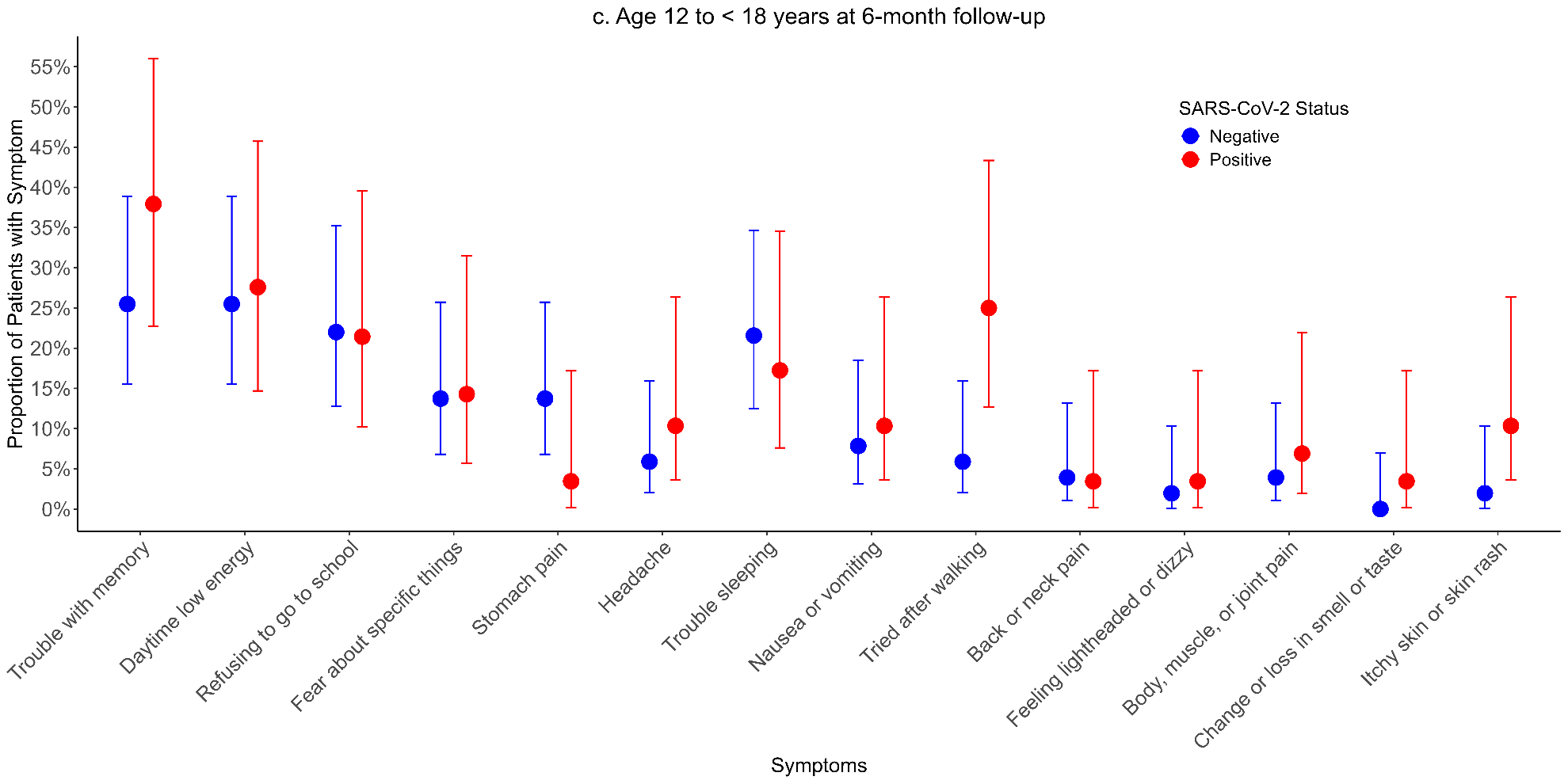


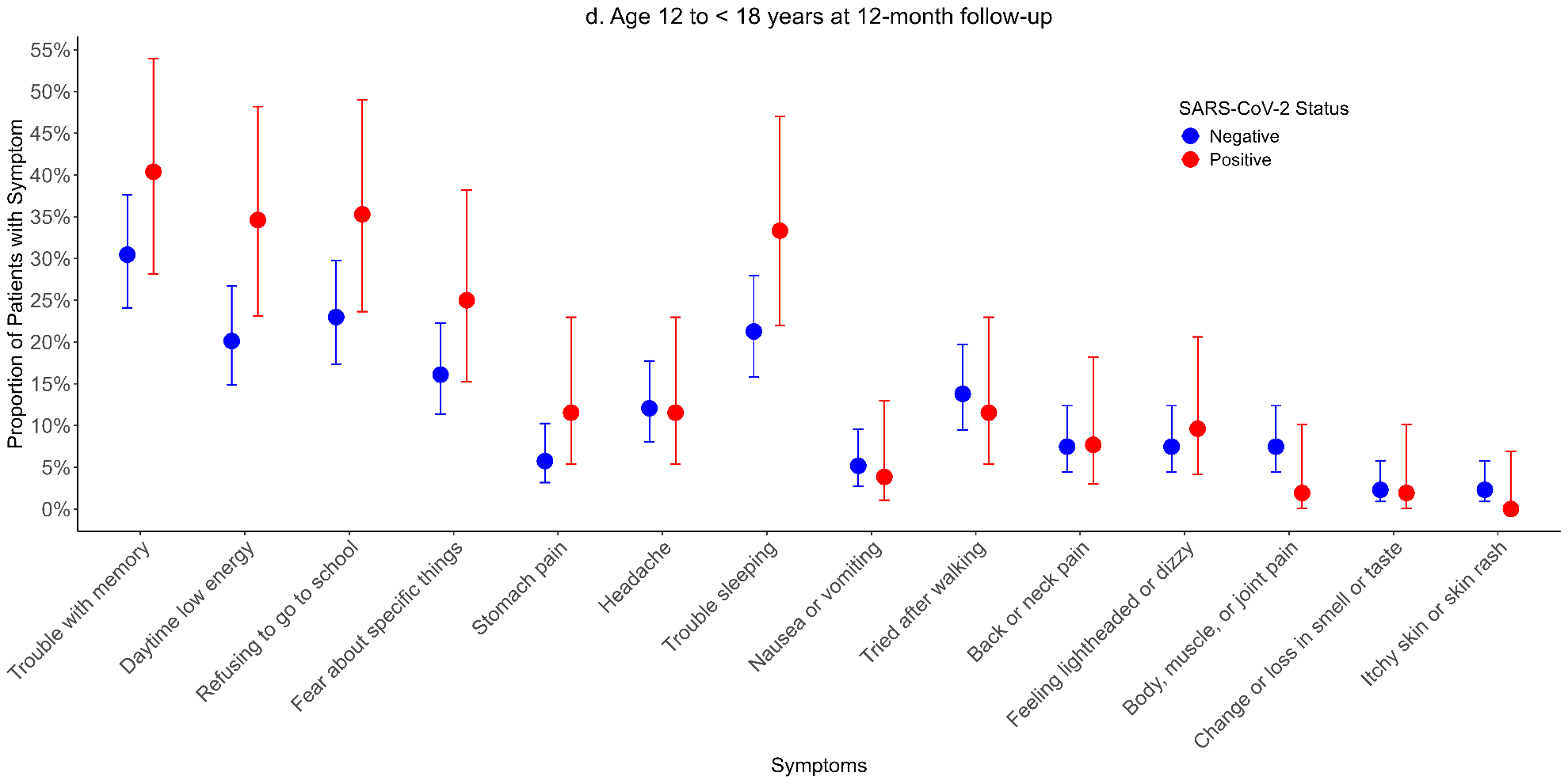


1. Gross RS, Thaweethai T, Kleinman LC, et al. Characterizing Long COVID in Children and Adolescents. *JAMA*. 2024;doi:10.1001/jama.2024.12747
